# A ‘what-if’ scenario: Nipah virus attacks pig trade chains in Thailand

**DOI:** 10.1186/s12917-020-02502-4

**Published:** 2020-08-24

**Authors:** Phrutsamon Wongnak, Weerapong Thanapongtharm, Worapan Kusakunniran, Sarattha Karnjanapreechakorn, Krittanat Sutassananon, Wantanee Kalpravidh, Kachen Wongsathapornchai, Anuwat Wiratsudakul

**Affiliations:** 1 Université Clermont Auvergne, INRAE, VetAgro Sup, UMR EPIA, 63122 Saint-Genès-Champanelle, France; 2 Université de Lyon, INRAE, VetAgro Sup, UMR EPIA, 69280 Marcy l’Etoile, France; 3 Department of Livestock Development (DLD), Bangkok, Thailand; 4 Faculty of Information and Communication Technology, Mahidol University, Nakhon Pathom, Thailand; 5 Food and Agriculture Organization of the United Nations, Global Emergency Centre for Transboundary Animal Diseases (ECTAD), Rome, Italy; 6 Food and Agriculture Organization of the United Nations, Regional Office for Asia and the Pacific, Bangkok, Thailand; 7 Department of Clinical Sciences and Public Health, and the Monitoring and Surveillance Center for Zoonotic Diseases in Wildlife and Exotic Animals, Faculty of Veterinary Science, Mahidol University, Nakhon Pathom, Thailand

**Keywords:** Animal movement, Nipah virus, Network modeling, Spatial risk

## Abstract

**Background:**

Nipah virus (NiV) is a fatal zoonotic agent that was first identified amongst pig farmers in Malaysia in 1998, in an outbreak that resulted in 105 fatal human cases. That epidemic arose from a chain of infection, initiating from bats to pigs, and which then spilled over from pigs to humans. In Thailand, bat-pig-human communities can be observed across the country, particularly in the central plain. The present study therefore aimed to identify high-risk areas for potential NiV outbreaks and to model how the virus is likely to spread. Multi-criteria decision analysis (MCDA) and weighted linear combination (WLC) were employed to produce the NiV risk map. The map was then overlaid with the nationwide pig movement network to identify the index subdistricts in which NiV may emerge. Subsequently, susceptible-exposed-infectious-removed (SEIR) modeling was used to simulate NiV spread within each subdistrict, and network modeling was used to illustrate how the virus disperses across subdistricts.

**Results:**

Based on the MCDA and pig movement data, 14 index subdistricts with a high-risk of NiV emergence were identified. We found in our infectious network modeling that the infected subdistricts clustered in, or close to the central plain, within a range of 171 km from the source subdistricts. However, the virus may travel as far as 528.5 km (R_0_ = 5).

**Conclusions:**

In conclusion, the risk of NiV dissemination through pig movement networks in Thailand is low but not negligible. The risk areas identified in our study can help the veterinary authority to allocate financial and human resources to where preventive strategies, such as pig farm regionalization, are required and to contain outbreaks in a timely fashion once they occur.

## Background

Nipah virus (NiV) is a negative-sense, single-stranded RNA virus. The virus belongs to the genus *Henipavirus*, family *Paramyxoviridae* [1]. In late September 1998, NiV first emerged amongst pig farmers and pigs in peninsular Malaysia [2]. The outbreak, which was associated with respiratory illness in pigs, and was first considered to be Japanese encephalitis [1]. Subsequently, a new virus closely related to Hendra virus, namely Nipah virus, was isolated [3]. By mid-June 1999, 265 individuals had fallen ill, 105 of which passed away (39.6% case-mortality rate). Consequently, over one million pigs were culled to control the outbreak [1, 3]. The NiV epidemic in Malaysia was subsequently found to have been initiated from bats to pigs, which then spilled over to humans [4]. In pigs, the virus is highly contagious and the morbidity rate may reach 100%, with a mortality rate of approximately 40%. The infected animals manifested either respiratory or neurological signs depending on age. A high proportion of pigs were infected asymptomatically [5], suggesting silent zoonotic transmission.

Three years later, a different strain of NiV emerged in Bangladesh and India [6, 7]. These genetically distinct strains were mainly driven by bat-to-human and human-to-human transmission, and the outbreaks appeared to occur annually [8–10]. Recently, another episode of NiV an outbreak was identified in India in May 2018, which resulted in 21 deaths among 23 confirmed cases (18 with laboratory results) [11]. Unsurprisingly, there was evidence suggesting that the fruit bat was likely to be the primary reservoir host responsible for the outbreaks [12]. Basically, NiV transmission was believed to occur through the consumption of foods contaminated with bat urine, such as date palm sap [13]. Pigs appeared to be an amplifying host for the 1998 NiV outbreaks in humans in Malaysia and Singapore, and direct contacts between bats and pigs were observed in the farm associated with the index case of the epidemic. Some of the evidence was piggeries placed under fruit trees, and half-consumed fruits were found within the piggeries [14]. To date, the spillover of NiV into pig and human populations has only been observed in Malaysia, Singapore, India, and Bangladesh [6, 8, 15, 16]. However, the genetic material of NiV in bats has been recovered from many more extended geographic locations, including Thailand [17, 18]. However, actual virus isolation has been limited.

**Fig. 1:**
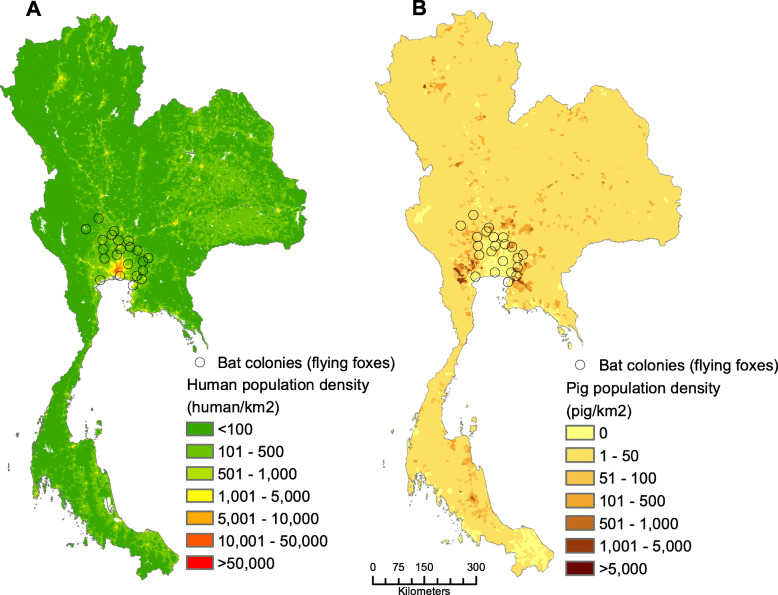
Human and pig population density, and the locations of bat roosting sites in Thailand, 2015. **a**. Human population density and the locations of flying fox colonies, and **b**. pig population density and the locations of flying fox colonies (the figure was originally created)

In Thailand, bat-pig-human communities can be observed across the country, particularly in the central plain, a combination of the central and eastern regions. The distribution density of humans and pigs in relation to bat roosting sites is illustrated in Fig. 1. This kind of environment ideally facilitates NiV emergence and dissemination. It has been suggested that pig farms with low biosecurity around Bangkok were at risk for NiV infection, as colonies of flying foxes (bats in the genus *Pteropus*) are located close by [19]. Flying foxes are the predominant bats found across the central plain, the area where human population density is high (232.07 individuals/km^2^ around the bat colonies). In addition, these bats are free to roam around the country, as bat hunting is completely prohibited, and bats are protected by the Wildlife Preservation and Protection Act, B.E. 2535 (1992). In the central plain, 22 colonies of flying foxes have been identified. Based on a satellite telemetry study, the foraging distances of these bats were estimated to be as far as 23.6 km [20]. Thirty-four food plants were consumed by the flying foxes, with the most common fruits recorded being mango, followed by banana and tamarind, respectively [20] and orchards of these fruits are found ubiquitously in the central plain of Thailand. Along their daily flying routes, the bats may occasionally visit some pig farms as previously evidenced in Malaysia [14]. Moreover, the genetic materials of both Malaysian and Bangladesh NiV strains have been detected in flying foxes (*Pteropus lylei*) [17].

The pig industry is an active and important business in Thailand. Pork meat is preferred in many Thai traditional dishes. Pig farming practice in Thailand is roughly divided into smallholder (a farm with less than 50 pigs) and large-scale (a farm with 50 pigs or more) farming systems, according to the Thai ‘Good Agricultural Practices for Pig Farms’ [21]. According to the Department of Livestock Development (DLD), a total of 10,191,784 pigs were recorded nationwide in 2017. These pigs were raised in 180,606 farms, and most farmers (93.6%) were considered smallholders [22]. In terms of pig types, the pigs in the farms can be either native, breeding, or fattening pigs. However, the majority of the smallholders raised native pigs, and their farms are generally located in rural areas to serve local consumption [21]. With limited budgets and resources, the biosecurity level in these small farms is relatively low [23]. Importantly, nearly half (49.1%) of all the pigs in the country are found in the central plain [22]. Considering the home ranges of the flying foxes, human density and number of pigs, NiV epidemics may occur at any time. Surprisingly, no trace of NiV infection has been reported in pig or human populations in Thailand. However, the dynamic transmission of the virus is worth exploring.

Mathematical modeling is a tool to forecast the magnitude of disease outbreaks, and epidemic models have been constructed for different diseases in different locations, for instance, foot-and-mouth disease in the US [24] and Mexico [25] as well as rabies in Australia [26]. In many cases, infectious diseases were found to spread via contact networks, driven by animal trade and movement [27].

As NiV has not yet impacted pig populations in Thailand, it is critically important to foresee how the disease could spread so that relevant preventive measures, such as pig farm regionalization, can be implemented in the right geographical locations. Moreover, the preparedness for possible epidemics is essential for future disease prevention and control. The present study, therefore, aimed to identify the high-risk areas in which NiV may emerge and then model how the virus is likely to spread along the pig trade network nationwide so that budgets and workforce can be allocated effectively once an outbreak occurs.

## Results

### Index subdistricts identification for the spread of NiV

**Fig. 2:**
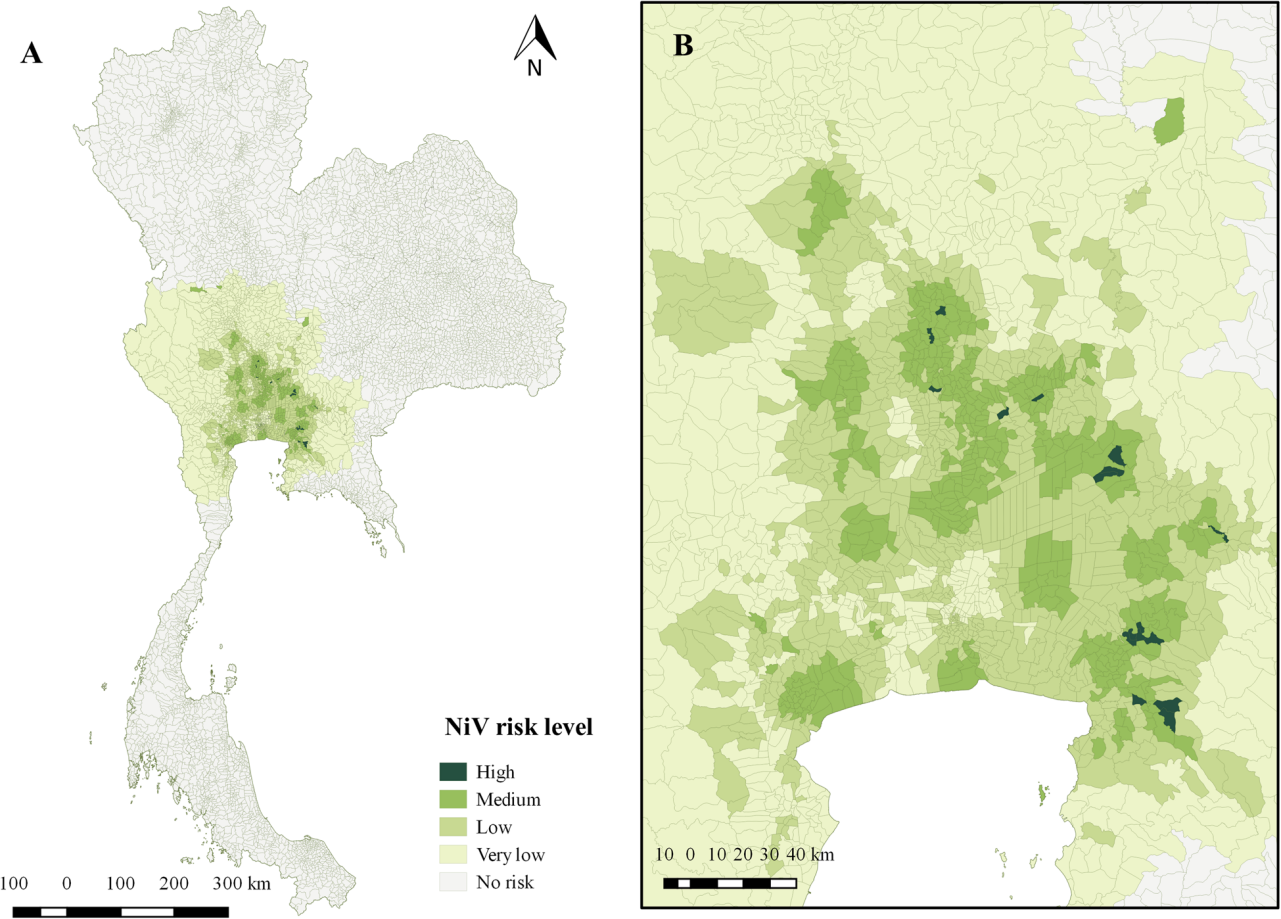
The spatial risk level of NiV occurrence in the central plain of Thailand identified by the MCDA method. **a**. The distribution of the study subdistricts on the map of Thailand, and **b**. The magnified map focusing on the central plain of Thailand (the figure was originally created)

Based on our MCDA method, we identified 17 high-risk subdistricts together with 764, 442, and 870 medium, low, and very low-risk subdistricts, respectively. The high-risk subdistricts (Table 1) were overlapped with a list of subdistricts where pig movement activity was known to occur. The risk map of NiV detection is depicted in Fig. 2. The complete list of NiV occurrence probability for the 2093 subdistricts analyzed in this study is given in Additional file 1.

**Table 1:** The list of high-risk subdistricts for NiV occurrence in Thailand

Geocode	Subdistrict	Province	Probability of NiV occurrence
240211	Sao Cha-Ngo	Chachoengsao	0.767383
170403	Bang Nam Chiao	Sing Buri	0.744118
260304	Asa	Nakhon Nayok	0.72053
200602	Na Phrathat	Chon Buri	0.716989
240107	Khlong Chuk Khachoe	Chachoengsao	0.709265
260307	Phikun Ok	Nakhon Nayok	0.707236
150306	Norasing	Ang Thong	0.67296
250106	Bang Boribun	Prachin Buri	0.659102
240212	Samet Nuea	Chachoengsao	0.637756
240106	Bang Phai	Chachoengsao	0.637532
200605	Na Roek	Chon Buri	0.631063
140908	Phra Kaeo	Phra Nakhon Si Ayutthaya	0.619647
200617	Na Wang Hin	Chon Buri	0.615036
241103	Bang Lao	Chachoengsao	0.614749
260308	Pa Kha	Nakhon Nayok	0.613451
150406	Bang Rakam	Ang Thong	0.612812
190502	Nong Khwai So	Saraburi	0.604876

In the pig movement database, the translocation of pigs was recorded in 3292 subdistricts nationwide, and specifically, pig movement was found in 14 out of 17 identified high-risk subdistricts. The three subdistricts without any pig movement records were Asa, Bang Lao, and Bang Rakam, and therefore these subdistricts were excluded from the list of index subdistricts.

### NiV transmission modeling

**Fig. 3:**
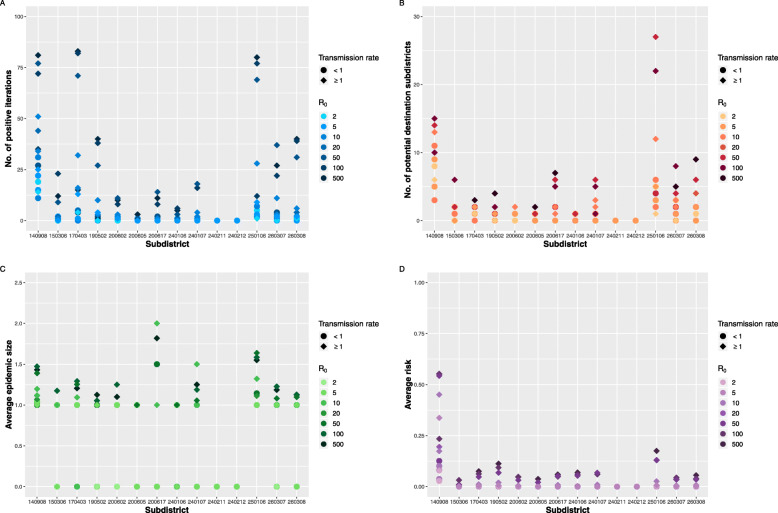
The NiV infectious modeling results, in which the disease transmission rate *β* and the basic reproduction number R_0_ were varied. **a**. Number of positive iterations observed from 100 iterations of each conditional simulation, **b**. Number of possible destination subdistricts that an index subdistrict of interest can spread the virus to, across 100 iterations, **c**. Average epidemic size calculated from the mean number of infected subdistricts an index subdistrict can infect in each iteration, and **d**. Average risk calculated from eq. 5. In total, 21,000 iterations were carried out to produce the results

In this study, we varied the values of transmission rate (*β*) and removal rate (*γ*) as the actual values from field observations are not available. Figure 3 illustrates how *β* and the basic reproduction number (R_0_) affect the NiV spreadability. Unsurprisingly, the more these two values increased, the more disease dissemination was observed. As shown in Fig. 3a, the probability that an index subdistrict may spread NiV outwards is around 1.00% (Inter-quartile range (IQR): 0.00–7.75%). However, a probability higher than 75% was observed in three subdistricts, including Phra Kaeo (140908), Bang Nam Chiao (170403), and Bang Boribun (Geocode 250,106). As shown in Fig. 3b, our models showed that at an R_0_ of 50, the virus might spread from Bang Boribun (Geocode 250,106) to another 27 destination subdistricts. The median number of possible infected subdistricts, across all iterations, originating from one initial subdistrict is 1 (IQR: 0–2). Focusing on the average epidemic size (Fig. 3c), 40.95% of all scenarios showed that NiV would never spread out. Interestingly, at an R_0_ of 2, the virus may diffuse to two other subdistricts, whereas the median of all average epidemic size observed in our models (excluding the epidemic size of 0) is 1 (IQR: 1–1.10). Indeed, the maximum epidemic size (unaveraged) was found in Phra Kaeo (Geocode 140,908), and Bang Boribun (Geocode 250,106) subdistricts at a value of 5. Figure 3d shows the average risk estimated for each initial subdistrict. In general, the risk was less than 0.13%. However, again higher risk was observed in Phra Kaeo (Geocode 140,908), and Bang Boribun (Geocode 250,106) subdistricts at a maximum of 0.55, and 0.18, respectively.

**Fig. 4:**
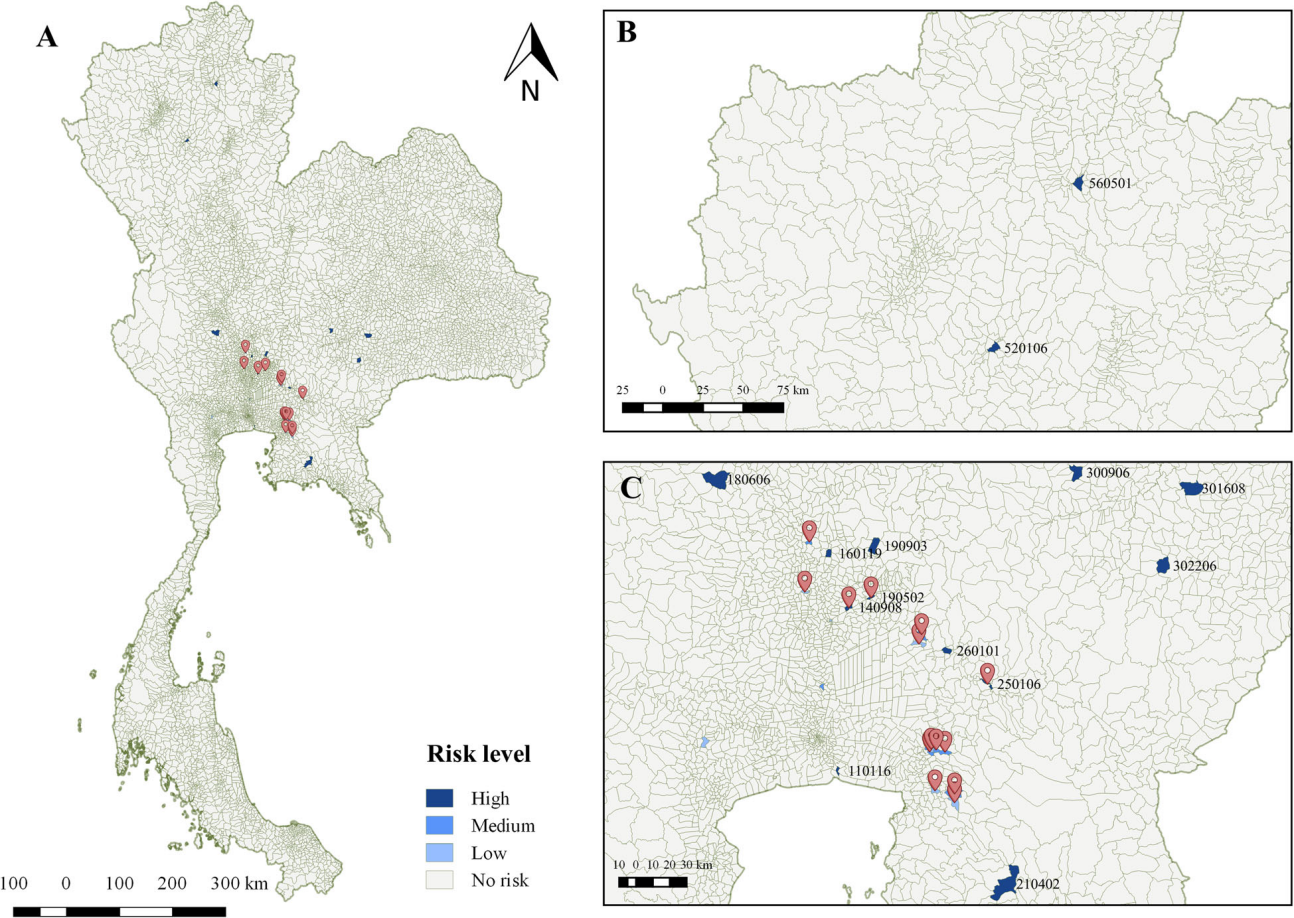
Geographical distribution of the index subdistricts (red pinpoints) and the destination subdistricts with different risk levels, based on R_0_ = 5 (*β* = 0.5, *γ* = 0.1). The risk level was categorized as ‘High’ (greater than 9.55 × 10^− 4^), ‘Medium’ (greater than 2.13 × 10^− 4^ to 9.55 × 10^− 4^), ‘Low’ (greater than 0 to 2.13 × 10^− 4^), and ‘No risk’ (risk = 0). **a**. The identified subdistricts are illustrated on the map of Thailand, **b**. A magnified map focusing on the northern part of Thailand and, **c**. A magnified map focusing on the central plain of Thailand where both index and destination subdistricts are concentrated (the figure was originally created)

The spatial distribution of NiV spread (R_0_ = 5.0) through the pig trade chain and its corresponding risk level from our model is illustrated in Fig. 4. All 14 index subdistricts could infect 14 other subdistricts, with Pra Kaew subdistrict in Phra Kakhon Si Ayutthaya (Geocode 140,908), as the highest infectious index subdistrict. This subdistrict may spread the virus to other 9 subdistricts. Most of the infected subdistricts are clustered in, or close to the central plain of Thailand, within a range of 171 km from their source subdistricts (see Fig. 4a and c). Interestingly, the subdistrict Pra Kaew could introduce NiV infected pigs much further to the Northern region, which are Chom Pu subdistrict in Lampang (Geocode 520,106; 440.8 km), and Dok Khamtai subdistrict in Phayao (Geocode 560,501; 528.5 km), respectively (Fig. 4b).

## Discussion

The present study primarily identified the spatial risk areas from which NiV may emerge, based on different contributing factors. The high-risk areas were then used to determine how NiV would spread across Thailand through the pig trade network. Our underlying assumption was that the virus would originate from the superimposed areas where both bat-pig spillover risk and concentrated pig trade activities were identified. Finally, disease transmission models were employed to illustrate how the virus would disseminate within and between subdistricts.

**Fig. 5:**
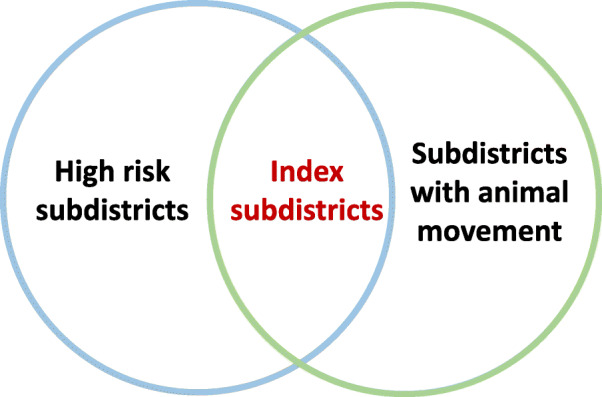
Identification of index subdistricts for the spread of NiV through pig movement in Thailand

Based on our spatial risk modeling, the number of identified high-risk areas was only 0.3% (7/2093) of all subdistricts analyzed in the model. This might be the reason why an outbreak of NiV has not occurred in the pig population in Thailand. Importantly, our results are in line with a previous study using another spatial risk identification method, namely, potential surface analysis (PSA) [19]. High-risk areas were identified in the central plain of the country (Fig. 2), where a high density of pigs and bat colonies have been observed [19]. In addition, the genetic material of NiV has been recovered in the same region [17, 18]. In our study, we mainly used the multi-criteria decision analysis (MCDA) method, which is a knowledge-based approach using existing knowledge to create decision rules and to integrate them with some potential factors to ultimately identify the risks [28, 29]. The MDCA methodology is an appropriate technique to be applied to this study, as the identification of relevant risk factors, their weights, and the way they increase the risk spatially may be defined more explicitly and thoroughly in using this approach [30]. The method has also been used in similar situations. For example, Paul and colleagues (2006) used MCDA to assess the suitability of areas for highly pathogenic avian influenza H5N1 in Thailand and applied this model to Cambodia [29].

As expected, outgoing pig movements were observed in 82.4% (14/17) of the identified high-risk subdistricts. The central plain was identified in a previous study as the main pig production area of Thailand, where different types of pig production systems are recognized [21]. The 14 subdistricts were then chosen to initiate NiV spread. In general, the probability that NiV can escape from the initial subdistricts is low except for the scenarios with extremely high R_0_ values (Fig. 3a). It is noteworthy that each index subdistrict can infect only a small number of other subdistricts (Fig. 3b). The virus was able to move to only one to two steps (Fig. 3c) before dying out, as no infected pigs were transported or no trade activities were observed in the dead-end subdistricts. Therefore, the overall risk of NiV transmission from these initial subdistricts is low (Fig. 3d) even with a high R_0_.

In this study, we also tried to explore what would happen with extreme situations, for example, using R_0_ values of 100 and 500. Surprisingly, we found that in many cases the risks were still low in such scenarios. Our models fully support the fact that the NiV has not emerged and spread in the pig populations in Thailand. Indeed, the DLD has conducted annual active surveillance for NiV detection in pigs in the country for more than 20 years, since the outbreak of NiV in Malaysia that emerged in 1998, and none has been detected. It is empirical and concrete evidence confirming that NiV has never emerged in our pig populations. Nonetheless, two high-risk initial subdistricts were identified in our models, including Phra Kaeo (Geocode 140,908) subdistrict, Phachi district, Phra Nakhon Si Ayutthaya province and Bang Boribun (Geocode 250,106) subdistrict, Mueang district, Prachinburi province. The former is a subdistrict with the second-highest number of outgoing pig transportation among the initial subdistricts. This explains the link between the number of animals moved and associated risk observed, as it has been suggested that the volume of animal movement is a key predictor of animal disease dissemination [31]. Moreover, the pigs exported from this subdistrict can travel quite far from the origin (Fig. 4b). The long-distance transportation of outgoing animal movements has been previously observed in different livestock species in Thailand, including cattle [32] and goats [33]. The socioeconomic motivations behind this trading behavior is worth exploring as moving animals across long distances is costly and time-consuming. There must be some convincing factors, for example, a higher selling rate compared to selling locally, that motivate animal traders to do so. To extend the study into this aspect, knowledge, and tools of social science and economics are needed. An insight into these motivations will help us in dealing with the behavior of the traders once an outbreak of infectious disease occurs.

We did face some potential limitations. Firstly, the pig movement data we used are the official animal movement data recorded electronically. This data recording system was designed to capture any inter-provincial livestock movements. However, the intra-provincial shipment of the animals is not recorded, and thus it is likely that we missed some hidden links that occurred within the provinces. This weakness was also pointed out in a previous study [34]. Thai veterinary authorities should reconstruct their procedures to include movement at the district or even subdistrict level. Secondly, we analyzed the spatial risk of NiV occurrence in less than one-third of all subdistricts in Thailand (2093/7416), as we focused on Thailand’s central plain, which had been previously identified as a high contact zone between pigs and flying foxes [19]. To expand the model to cover the whole country, a field study to identify the current locations of bat colonies nationwide should be undertaken, together with increased data collection on other factors needed in the spatial risk model construction. However, the present study initially carried out a risk analysis of one of the hotspots identified in a previous study [35] using a more sophisticated analytical technique. Our methods could be applied more widely once more data are available. Thirdly, local transmission was not considered in our model. We exclusively focused on how NiV spread along the trade chain. Indeed, the virus may disperse locally by other means. A more sophisticated modeling approach, such as an individual-based system at an animal or farm level, is recommended. Fourthly, we did not include other factors that may influence how NiV spreads in the pig population, such as meteorological effects and seasonality. However, we intended to produce an initial model focusing on the spread of the virus through the pig movement. A future study, including other contributing factors, is recommended. Fifthly, the β and γ values of NiV transmission are not known. We used different values of these parameters to explore the likelihood of disease spread. If we have better knowledge of these parameters in the future, the model will be more accurate and suitable for an outbreak situation. Sixthly, the epidemiological model itself always comes with some general limitations. In the MCDA framework, as it is a knowledge-based approach, the subjectivity of the method derived from the involvement of experts’ opinions might be a significant limitation. Nonetheless, this approach is still practical, especially in the case that actual field data is unavailable, as in our case. In the SEIR model, the population is assumed to be homogeneously mixed while the actual pig population in the subdistrict is further divided into the farm level. However, the SEIR model is still one of the best choices when we need to work with big data as it is less time-consuming compared to other more complex models like an individual-based framework. The last and the most important limitation found in our study was that Thailand has never experienced an NiV epidemic. The NiV outbreak in pig populations in Malaysia has been the only recognized attack in veterinary history so far. To contain that particular devastating epidemic, millions of pigs were immediately culled [1, 4]. It was too rapid to observe any epidemiological characteristics of NiV propagation. Consequently, we need to vary different epidemic parameters to visualize different outbreak scenarios that are likely to occur. However, it is not possible to validate our assumptions. The best we can do is to quantify the risk and promptly prepare for the potential outbreaks. Nonetheless, our simulation framework is exploitable as a baseline model to examine the effectiveness of control strategies, and to suggest some practical contingency plans. In addition, our model is able to identify the areas where pig farming practices should be improved to prevent the occurrence of an NiV outbreak. In this study, we produced a simulation framework that is usable as a baseline structure for any interventional modeling. We acknowledge here that our model did not consider the nature of the pig movement. A future study may simulate animal movement according to the intended purpose, such as to a slaughterhouse and include relevant parameters for the spread of the virus. This will make the model more realistic, but it requires a valid set of field data to do so.

## Conclusions

The risk of NiV dissemination through pig movement networks in Thailand is low but not negligible as we have a perfect environment for NiV emergence in the country. The risk areas identified in our study may help veterinary authorities to allocate financial and human resources to where preventive strategies, such as pig farm regionalization are required, and to contain outbreaks in a timely fashion once they occur.

## Methods

### Index subdistricts identification for the spread of NiV

The present study focused on the transmission of NiV at the subdistrict level, the smallest administrative unit in Thailand with standard geocodes [36]. We focused on 2093 subdistricts located in the central plain of Thailand, where bat colonies and pig farms are highly concentrated. The spatial risk of NiV emergence was assessed using the multi-criteria decision analysis (MCDA) method, and the results were published previously [35]. In this study, we sought to expand on those results. Briefly, we invited 20 experts in epidemiology, virology, pig farming systems, and bat ecology to attend a workshop for the decision-making process. The experts identified i) spatial risk factors of NiV transmission including bat preferred areas, distance to the nearest bat colony, pig population density, distance to the nearest forest, distance to the nearest orchard, distance to the nearest water body, and human population density, ii) the experts identified the association between the values of each factor and the suitability of NiV distribution by using fuzzy membership functions, and iii) technique. The details of all steps are explained in [35]. Subsequently, the geometric information system (GIS) method with a weighted linear combination (WLC) was used to combine all spatial risk layers to generate a final estimated map. The risk was divided into five levels according to the probability of disease occurrence, namely very high (0.8–1), high (0.6–0.8), medium (0.4–0.6), low (0.2–0.4), and very low (0.0–0.2). The high-risk subdistricts (> 0.6) were then overlaid on the subdistricts with pig movement activities, based on the national animal movement database of the DLD, to identify the subdistricts that NiV may initiate the transmission through the pig movement network (Fig. 5).

### NiV transmission modeling

#### Within subdistricts

**Fig. 6:**
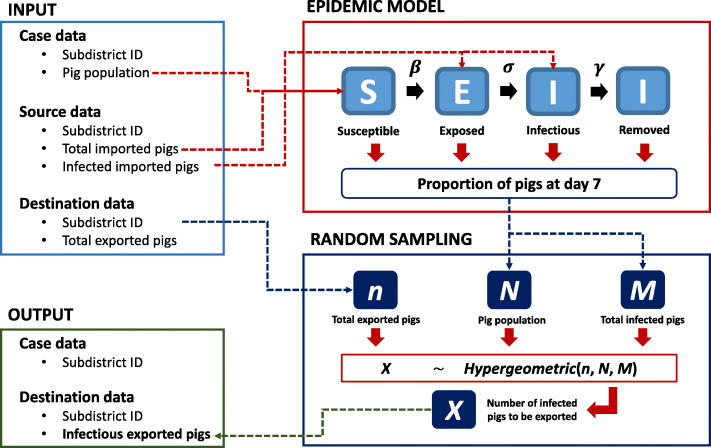
Conceptual framework of NiV transmission within a subdistrict

An official database of pig population at the subdistrict level in Thailand in 2017 was obtained from the DLD [22]. The data was then used as a baseline in our NiV infectious modeling within each subdistrict. An index subdistrict was chosen from the list prepared from the previous step. An NiV infected pig was then introduced into the selected subdistrict. A Susceptible-Exposed-Infectious-Removed (SEIR) model was employed, as shown in eq. 1. We varied the parameters to deal with uncertainty by calculating the basic reproduction number R_0_ (*β*/*γ*), as shown in Table 2. Note that only the R_0_ > 1 was used, as this allows the disease to spread [37]. In this model, we followed a latent period (*σ*) of 6 days based on a previous study [37]. The model was constructed on a weekly basis. At the end of the week, a specific number of pigs were designated to a subdistrict that was randomly chosen from the destinations list recorded in the pig movement database. The within subdistrict transmission is conceptualized as demonstrated in Fig. 6.

1 $$ {\displaystyle \begin{array}{c}\frac{\mathrm{dS}}{\mathrm{dt}}=-\frac{\beta \mathrm{SI}}{\mathrm{N}}\\ {}\frac{\mathrm{dE}}{\mathrm{dt}}=\frac{\beta \mathrm{SI}}{\mathrm{N}}-\sigma \mathrm{E}\\ {}\frac{\mathrm{dI}}{\mathrm{dt}}=\sigma \mathrm{E}-\gamma \mathrm{I}\\ {}\frac{\mathrm{dR}}{\mathrm{dt}}=\gamma \mathrm{I}\end{array}} $$

**Table 2:** The R_0_ values varied from different *β* and *γ* values. The values higher than 1 were used in the NiV spread simulation

Transmission rate (***β***)	Removal rate (***γ***)				
	0.01	0.05	0.1	0.5	1
**0.05**	5	1	0.5	0.1	0.05
**0.1**	10	2	1	0.2	0.1
**0.5**	50	10	5	1	0.5
**1**	100	20	10	2	1
**5**	500	100	50	10	5

, where S = Susceptible, E = Exposed, I = Infectious, R = Removed, *β =* transmission rate, γ = removal rate and *σ* = latent period. The model is governed by a differential ordinary equation.

The susceptible (S) is the subpopulation without immunity which is capable of being infected, exposed (E) is the population that is already infected, but not yet infectious (unable to spread the virus), infectious (I) develops from exposed individuals after a certain period (latent period) They can spread the virus to susceptible individuals and removed (R) is defined here as pigs that die or recover from the infection.

### Between subdistricts

**Fig. 7:**
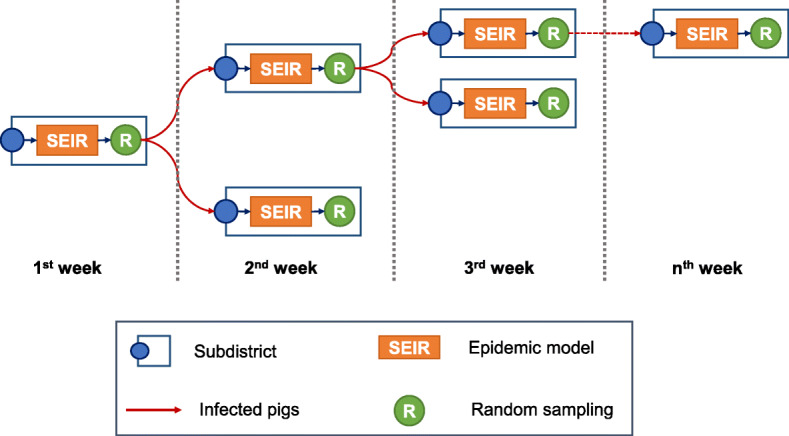
Conceptual framework of NiV transmission between subdistricts

The between subdistrict modeling of the NiV spread is conceptualized in Fig. 7. To model pig movement from a source subdistrict i (*S*_*i*_), a list of modeled destination subdistricts (*M*) and the number of transported pigs (*N*) are generated. The number of weekly pig movements of *S*_*i*_ (*n*_*i*_) is generated with the following Poisson distribution:

2 $$ {n}_i\sim \mathrm{Pois}\left({\lambda}_i\right) $$

; where *λ*_*i*_ is the mean number of weekly pig movements of *S*_*i*_ derived from the animal movement dataset. Let total *j* subdistricts (D) be the members of a set of all possible destination subdistricts of *S*_*i*_ (*D*_*P*_). The probability of *D*_*j*_ being selected as a destination subdistrict in the modeled pig movement (*p*_*j*_) is calculated as:

3 $$ \mathrm{pj}=\frac{{\mathrm{f}}_{\mathrm{j}}}{\mathrm{F}} $$

; where *f*_*j*_ is an annual pig movement frequency from *S*_*i*_ to *D*_*j*_, and *F* is the total number of annual pig movement frequency of *S*_*i*_ to all destinations. Then, total *n*_*i*_ destination subdistricts are randomly selected from *D*_*P*_ as members of M with their respective probabilities *p*. Finally, the number of pigs transported (*N*_*k*_) from *S*_*i*_ to a subdistrict of destination k (*M*_*k*_) is generated following a normal distribution:

4 $$ {\mathrm{N}}_{\mathrm{k}}\sim \mathrm{Normal}\left({\overline{\mathrm{X}}}_{\mathrm{k}},{s}_{\mathrm{k}}\right) $$

; where *X̅*_*k*_ and *s*_*k*_ are the mean and standard deviation of the number of pigs transported from *S*_*i*_ to *M*_*k*_ per movement, according to the actual animal movement dataset.

We repeated the whole process for all subdistricts listed as potentially high-risk areas for the NiV spread. In each index subdistrict, we recorded four main outputs; (i) number of positive iterations in which the NiV can spread out from the index subdistricts, (ii) number of potential destination subdistricts, the aggregation of all possible subdistricts that NiV reached across iterations, (iii) average epidemic size which is the mean number of infected subdistricts resulting from an index subdistrict in each iteration, and (iv) average risk (π) which is calculated from the following equation:

5 $$ \pi =\frac{1}{N_{\pi }}\sum \limits_{i=1}^I\sum \limits_{j=1}^J{\pi}_{ij},{\pi}_{ij}>0 $$

; where *I* and *J* denotes the number of infected subdistricts resulting from an index subdistrict, and the number of total iterations, respectively. While *π*_*ij*_ is the risk of subdistrict i of the j^th^ iteration, and *N*_*π*_ is the total number of *π*_*ij*_ > 0.

We used programming language R version 3.5.1 in the spatial risk analysis and the within subdistrict modeling of NiV spread. The between subdistrict modeling of pig trade and the NiV dissemination were mechanized by Visual studio code with python version 3.6.8. The pig movement and disease transmission were stochastically simulated in 100 iterations each. In total, 21,000 iterations were carried out to produce the results. The risk maps were visualized with QGIS application version 2.18.24.

## Supplementary information

**Additional file 1: Table S1.** The list of high risk subdistricts for NiV occurrence at subdistrict level. **Table S2.** The list of medium risk subdistricts for NiV occurrence at subdistrict level. **Table S3.** The list of low risk subdistricts for NiV occurrence at subdistrict level. **Table S4.** The list of very low risk subdistricts for NiV occurrence at subdistrict level.

## Data Availability

The data that support the findings of this study are available from the Department of Livestock Development, but restrictions apply to the availability of these data, which were used under license for the current study, and so are not publicly available. Data are, however, available from the authors upon reasonable request and with permission of the Department of Livestock Development.

## Abbreviations

BE: Buddhist Era
DLD: Department of Livestock Development
GIS: Geographic information system
IQR: Inter-quartile range
MCDA: Multi-criteria decision analysis
NiV: Nipah virus
PSA: Potential surface analysis
RNA: Ribonucleic acid
SEIR: Susceptible-exposed-infectious-removed
US: United States
WLC: Weighted linear combination
