# Supplementary material for: A ‘what-if’ scenario: Nipah virus attacks pig trade chains in Thailand
Source: BMC Vet Res. 2020 Aug 24;16:300. doi: 10.1186/s12917-020-02502-4 (PMC7446211; doi:10.1186/s12917-020-02502-4)
Supplement: Supplementary file 1 — Additional file 1: Table S1. The list of high risk subdistricts for NiV occurrence at subdistrict level. Table S2. The list of medium risk subdistricts for NiV occurrence at subdistrict level. Table S3. The list of low risk subdistricts for NiV occurrence at subdistrict level. Table S4. The list of very low risk subdistricts for NiV occurrence at subdistrict level. [file 12917_2020_2502_MOESM1_ESM.docx]

**Table S1** The list of high risk subdistricts for NiV occurrence at subdistrict level

| **Geocode** | **Name Of The Subdistrict** | **Province** | **Probability of NiV occurrence** |
| --- | --- | --- | --- |
| 140908 | Phra Kaeo | Phra Nakhon Si Ayutthaya | 0.619647 |
| 150306 | Norasing | Ang Thong | 0.67296 |
| 150406 | Bang Rakam | Ang Thong | 0.612812 |
| 170403 | Bang Nam Chiao | Sing Buri | 0.744118 |
| 190502 | Nong Khwai So | Saraburi | 0.604876 |
| 200602 | Na Phrathat | Chon Buri | 0.716989 |
| 200605 | Na Roek | Chon Buri | 0.631063 |
| 200617 | Na Wang Hin | Chon Buri | 0.615036 |
| 240106 | Bang Phai | Chachoengsao | 0.637532 |
| 240107 | Khlong Chuk Khachoe | Chachoengsao | 0.709265 |
| 240211 | Sao Cha-Ngo | Chachoengsao | 0.767383 |
| 240212 | Samet Nuea | Chachoengsao | 0.637756 |
| 241103 | Bang Lao | Chachoengsao | 0.614749 |
| 250106 | Bang Boribun | Prachin Buri | 0.659102 |
| 260304 | Asa | Nakhon Nayok | 0.72053 |
| 260307 | Phikun Ok | Nakhon Nayok | 0.707236 |
| 260308 | Pa Kha | Nakhon Nayok | 0.613451 |

**Table S2** The list of medium risk subdistricts for NiV occurrence at subdistrict level

| **Geocode** | **Name of the subdistrict** | **Province** | **Probability of NiV occurrence** |
| --- | --- | --- | --- |
| 100301 | Krathum Rai | Bangkok | 0.403575 |
| 100302 | Nong Chok | Bangkok | 0.498161 |
| 100303 | Khlong Sip | Bangkok | 0.512947 |
| 100304 | Khlong Sip Song | Bangkok | 0.478615 |
| 100305 | Khok Faet | Bangkok | 0.463037 |
| 100306 | Khu Fung Nuea | Bangkok | 0.494228 |
| 100307 | Lam Phak Chi | Bangkok | 0.436884 |
| 100308 | Lam Toiting | Bangkok | 0.413978 |
| 101002 | Saen Saep | Bangkok | 0.41251 |
| 104602 | Sam Wa Tawan Ok | Bangkok | 0.466601 |
| 104604 | Sai Kong Din | Bangkok | 0.422648 |
| 104605 | Sai Kong Din Tai | Bangkok | 0.468815 |
| 104902 | Thung Khru | Bangkok | 0.436794 |
| 110104 | Thai Ban | Samut Prakan | 0.413298 |
| 110111 | Bang Prong | Samut Prakan | 0.427626 |
| 110113 | Bang Duan | Samut Prakan | 0.480421 |
| 110208 | Khlong Niyom Yattra | Samut Prakan | 0.456213 |
| 110401 | Talat | Samut Prakan | 0.454757 |
| 110405 | Bang Ya Phraek | Samut Prakan | 0.407996 |
| 110406 | Bang Hua Suea | Samut Prakan | 0.414709 |
| 110411 | Bang Krasop | Samut Prakan | 0.408394 |
| 110413 | Song Khanong | Samut Prakan | 0.443967 |
| 110501 | Na Kluea | Samut Prakan | 0.427367 |
| 110502 | Ban Khlong Suan | Samut Prakan | 0.419349 |
| 110503 | Laem Fa Pha | Samut Prakan | 0.40508 |
| 110504 | Pak Khlong Bang Pla Kot | Samut Prakan | 0.445048 |
| 110505 | Nai Khlong Bang Pla Kot | Samut Prakan | 0.442369 |
| 120505 | Khun Si | Nonthaburi | 0.442597 |
| 130403 | Bueng Ka Sam | Pathum Thani | 0.434965 |
| 130406 | Sala Khru | Pathum Thani | 0.413235 |
| 130407 | Noppharat | Pathum Thani | 0.484332 |
| 130503 | Khu Bang Luang | Pathum Thani | 0.443336 |
| 130504 | Khu Khwang | Pathum Thani | 0.483282 |
| 130506 | Bo Ngoen | Pathum Thani | 0.442454 |
| 130604 | Lam Luk Ka | Pathum Thani | 0.436854 |
| 130605 | Bueng Thong Lang | Pathum Thani | 0.474816 |
| 130606 | Lam Sai | Pathum Thani | 0.45928 |
| 130608 | Phuet Udom | Pathum Thani | 0.441117 |
| 130701 | Bang Toei | Pathum Thani | 0.485368 |
| 130702 | Khlong Khwai | Pathum Thani | 0.522961 |
| 130703 | Sam Khok | Pathum Thani | 0.412067 |
| 130704 | Krachaeng | Pathum Thani | 0.406542 |
| 130705 | Bang Pho Nuea | Pathum Thani | 0.40969 |
| 130707 | Ban Pathum | Pathum Thani | 0.438709 |
| 130708 | Ban Ngio | Pathum Thani | 0.493625 |
| 130709 | Chiang Rak Noi | Pathum Thani | 0.453353 |
| 130710 | Bang Krabue | Pathum Thani | 0.530798 |
| 130711 | Thai Ko | Pathum Thani | 0.498861 |
| 140102 | Kamang | Phra Nakhon Si Ayutthaya | 0.482417 |
| 140103 | Ho Rattana Chai | Phra Nakhon Si Ayutthaya | 0.412034 |
| 140104 | Hua Ro | Phra Nakhon Si Ayutthaya | 0.474982 |
| 140105 | Tha Wasukri | Phra Nakhon Si Ayutthaya | 0.439572 |
| 140109 | Samphao Lom | Phra Nakhon Si Ayutthaya | 0.457856 |
| 140110 | Suan Phrik | Phra Nakhon Si Ayutthaya | 0.44218 |
| 140111 | Khlong Takhian | Phra Nakhon Si Ayutthaya | 0.492849 |
| 140113 | Han Tra | Phra Nakhon Si Ayutthaya | 0.438404 |
| 140115 | Ban Mai | Phra Nakhon Si Ayutthaya | 0.46121 |
| 140116 | Ban Ko | Phra Nakhon Si Ayutthaya | 0.45775 |
| 140117 | Khlong Suan Phlu | Phra Nakhon Si Ayutthaya | 0.442136 |
| 140118 | Khlong Sa Bua | Phra Nakhon Si Ayutthaya | 0.464877 |
| 140119 | Ko Rian | Phra Nakhon Si Ayutthaya | 0.518003 |
| 140120 | Ban Pom | Phra Nakhon Si Ayutthaya | 0.418451 |
| 140121 | Ban Run | Phra Nakhon Si Ayutthaya | 0.443775 |
| 140202 | Champa | Phra Nakhon Si Ayutthaya | 0.41954 |
| 140203 | Tha Luang | Phra Nakhon Si Ayutthaya | 0.443719 |
| 140205 | Sala Loi | Phra Nakhon Si Ayutthaya | 0.46327 |
| 140207 | Pho En | Phra Nakhon Si Ayutthaya | 0.424138 |
| 140209 | Nong Khanak | Phra Nakhon Si Ayutthaya | 0.419491 |
| 140210 | Tha Chao Sanuk | Phra Nakhon Si Ayutthaya | 0.409317 |
| 140301 | Nakhon Luang | Phra Nakhon Si Ayutthaya | 0.492109 |
| 140302 | Tha Chang | Phra Nakhon Si Ayutthaya | 0.431518 |
| 140303 | Bo Phong | Phra Nakhon Si Ayutthaya | 0.407918 |
| 140305 | Pak Chan | Phra Nakhon Si Ayutthaya | 0.411624 |
| 140306 | Bang Rakam | Phra Nakhon Si Ayutthaya | 0.474805 |
| 140307 | Bang Phrakhru | Phra Nakhon Si Ayutthaya | 0.442081 |
| 140308 | Mae La | Phra Nakhon Si Ayutthaya | 0.451214 |
| 140310 | Khlong Sakae | Phra Nakhon Si Ayutthaya | 0.437438 |
| 140311 | Sam Thai | Phra Nakhon Si Ayutthaya | 0.423061 |
| 140312 | Phra Non | Phra Nakhon Si Ayutthaya | 0.413154 |
| 140401 | Bang Sai | Phra Nakhon Si Ayutthaya | 0.44493 |
| 140402 | Bang Phli | Phra Nakhon Si Ayutthaya | 0.481066 |
| 140403 | Sanam Chai | Phra Nakhon Si Ayutthaya | 0.489755 |
| 140404 | Ban Paeng | Phra Nakhon Si Ayutthaya | 0.436607 |
| 140407 | Khae Ok | Phra Nakhon Si Ayutthaya | 0.414386 |
| 140408 | Khae Tok | Phra Nakhon Si Ayutthaya | 0.431005 |
| 140410 | Krachaeng | Phra Nakhon Si Ayutthaya | 0.411312 |
| 140413 | Ho Mok | Phra Nakhon Si Ayutthaya | 0.402282 |
| 140415 | Kok Kaeo Burapha | Phra Nakhon Si Ayutthaya | 0.401155 |
| 140416 | Mai Tra | Phra Nakhon Si Ayutthaya | 0.506942 |
| 140417 | Ban Ma | Phra Nakhon Si Ayutthaya | 0.461006 |
| 140419 | Ratchakhram | Phra Nakhon Si Ayutthaya | 0.518462 |
| 140420 | Chang Yai | Phra Nakhon Si Ayutthaya | 0.492959 |
| 140421 | Pho Taeng | Phra Nakhon Si Ayutthaya | 0.454114 |
| 140423 | Khok Chang | Phra Nakhon Si Ayutthaya | 0.510698 |
| 140503 | Sai Noi | Phra Nakhon Si Ayutthaya | 0.420293 |
| 140515 | Bang Chani | Phra Nakhon Si Ayutthaya | 0.438108 |
| 140601 | Ban Len | Phra Nakhon Si Ayutthaya | 0.409385 |
| 140602 | Chiang Rak Noi | Phra Nakhon Si Ayutthaya | 0.407642 |
| 140603 | Ban Pho | Phra Nakhon Si Ayutthaya | 0.525909 |
| 140604 | Ban Krot | Phra Nakhon Si Ayutthaya | 0.444467 |
| 140605 | Bang Krasan | Phra Nakhon Si Ayutthaya | 0.433481 |
| 140606 | Khlong Chik | Phra Nakhon Si Ayutthaya | 0.438784 |
| 140608 | Wat Yom | Phra Nakhon Si Ayutthaya | 0.422183 |
| 140609 | Bang Pradaeng | Phra Nakhon Si Ayutthaya | 0.451089 |
| 140610 | Sam Ruean | Phra Nakhon Si Ayutthaya | 0.454182 |
| 140611 | Ko Koet | Phra Nakhon Si Ayutthaya | 0.464155 |
| 140612 | Ban Phlap | Phra Nakhon Si Ayutthaya | 0.439857 |
| 140613 | Ban Paeng | Phra Nakhon Si Ayutthaya | 0.420445 |
| 140614 | Khung Lan | Phra Nakhon Si Ayutthaya | 0.445548 |
| 140615 | Taling Chan | Phra Nakhon Si Ayutthaya | 0.442193 |
| 140616 | Ban Sang | Phra Nakhon Si Ayutthaya | 0.439808 |
| 140617 | Talat Kriap | Phra Nakhon Si Ayutthaya | 0.439824 |
| 140618 | Khanon Luang | Phra Nakhon Si Ayutthaya | 0.521244 |
| 140701 | Bang Pahan | Phra Nakhon Si Ayutthaya | 0.464504 |
| 140702 | Khayai | Phra Nakhon Si Ayutthaya | 0.430417 |
| 140703 | Bang Duea | Phra Nakhon Si Ayutthaya | 0.46984 |
| 140704 | Sao Thong | Phra Nakhon Si Ayutthaya | 0.530648 |
| 140705 | Thang Klang | Phra Nakhon Si Ayutthaya | 0.540667 |
| 140706 | Bang Phloeng | Phra Nakhon Si Ayutthaya | 0.461081 |
| 140707 | Han Sang | Phra Nakhon Si Ayutthaya | 0.497913 |
| 140708 | Bang Nang Ra | Phra Nakhon Si Ayutthaya | 0.48543 |
| 140709 | Tanim | Phra Nakhon Si Ayutthaya | 0.51019 |
| 140711 | Ban Ma | Phra Nakhon Si Ayutthaya | 0.424744 |
| 140712 | Khwan Mueang | Phra Nakhon Si Ayutthaya | 0.454971 |
| 140713 | Ban Li | Phra Nakhon Si Ayutthaya | 0.414065 |
| 140714 | Pho Sam Ton | Phra Nakhon Si Ayutthaya | 0.476525 |
| 140715 | Phut Lao | Phra Nakhon Si Ayutthaya | 0.404993 |
| 140716 | Tan En | Phra Nakhon Si Ayutthaya | 0.490712 |
| 140904 | Nong Nam Sai | Phra Nakhon Si Ayutthaya | 0.474364 |
| 140905 | Don Ya Nang | Phra Nakhon Si Ayutthaya | 0.439533 |
| 141004 | Phraya Banlue | Phra Nakhon Si Ayutthaya | 0.455103 |
| 141005 | Singhanat | Phra Nakhon Si Ayutthaya | 0.487648 |
| 141007 | Khlong Phraya Banlue | Phra Nakhon Si Ayutthaya | 0.415831 |
| 141102 | Bo Ta Lo | Phra Nakhon Si Ayutthaya | 0.421135 |
| 141105 | Sanap Thuep | Phra Nakhon Si Ayutthaya | 0.431215 |
| 141106 | Phayom | Phra Nakhon Si Ayutthaya | 0.575184 |
| 141401 | Khan Ham | Phra Nakhon Si Ayutthaya | 0.420372 |
| 141402 | Ban Chang | Phra Nakhon Si Ayutthaya | 0.452662 |
| 141404 | Ban Hip | Phra Nakhon Si Ayutthaya | 0.408499 |
| 141406 | Uthai | Phra Nakhon Si Ayutthaya | 0.436966 |
| 141410 | Thanu | Phra Nakhon Si Ayutthaya | 0.457467 |
| 141411 | Khao Mao | Phra Nakhon Si Ayutthaya | 0.421152 |
| 141501 | Hua Phai | Phra Nakhon Si Ayutthaya | 0.531073 |
| 141502 | Kathum | Phra Nakhon Si Ayutthaya | 0.442778 |
| 141503 | Maharat | Phra Nakhon Si Ayutthaya | 0.548006 |
| 141504 | Namtao | Phra Nakhon Si Ayutthaya | 0.517843 |
| 141506 | Rong Chang | Phra Nakhon Si Ayutthaya | 0.466646 |
| 141507 | Chao Pluk | Phra Nakhon Si Ayutthaya | 0.493061 |
| 141508 | Phit Phian | Phra Nakhon Si Ayutthaya | 0.482371 |
| 141510 | Ban Khwang | Phra Nakhon Si Ayutthaya | 0.476108 |
| 141511 | Tha To | Phra Nakhon Si Ayutthaya | 0.418583 |
| 141512 | Ban Mai | Phra Nakhon Si Ayutthaya | 0.432504 |
| 141601 | Ban Phraek | Phra Nakhon Si Ayutthaya | 0.404385 |
| 141602 | Ban Mai | Phra Nakhon Si Ayutthaya | 0.409635 |
| 141603 | Sam Phaniang | Phra Nakhon Si Ayutthaya | 0.482304 |
| 141604 | Khlong Noi | Phra Nakhon Si Ayutthaya | 0.413605 |
| 150102 | Bang Kaeo | Ang Thong | 0.41921 |
| 150103 | Sala Daeng | Ang Thong | 0.44336 |
| 150104 | Pa Ngio | Ang Thong | 0.470143 |
| 150105 | Ban Hae | Ang Thong | 0.467287 |
| 150106 | Talat Kruat | Ang Thong | 0.468125 |
| 150108 | Ban It | Ang Thong | 0.400487 |
| 150109 | Hua Phai | Ang Thong | 0.487264 |
| 150110 | Champa Lo | Ang Thong | 0.449562 |
| 150111 | Pho Sa | Ang Thong | 0.474538 |
| 150112 | Ban Ri | Ang Thong | 0.431175 |
| 150113 | Khlong Wua | Ang Thong | 0.430918 |
| 150114 | Yan Sue | Ang Thong | 0.47778 |
| 150201 | Chorakhe Rong | Ang Thong | 0.446375 |
| 150202 | Chaiyaphum | Ang Thong | 0.533392 |
| 150203 | Chaiyarit | Ang Thong | 0.437007 |
| 150204 | Thewarat | Ang Thong | 0.585151 |
| 150205 | Ratsathit | Ang Thong | 0.543912 |
| 150206 | Chaiyo | Ang Thong | 0.584418 |
| 150207 | Lak Fa | Ang Thong | 0.475395 |
| 150208 | Chawai | Ang Thong | 0.460798 |
| 150301 | Bang Pla Kot | Ang Thong | 0.470528 |
| 150303 | Sai Thong | Ang Thong | 0.471929 |
| 150304 | Rong Chang | Ang Thong | 0.408017 |
| 150401 | Ang Kaeo | Ang Thong | 0.514041 |
| 150402 | Inthapramun | Ang Thong | 0.54168 |
| 150403 | Bang Phlap | Ang Thong | 0.572122 |
| 150404 | Nong Mae Kai | Ang Thong | 0.409416 |
| 150407 | Pho Rang Nok | Ang Thong | 0.596721 |
| 150408 | Ongkharak | Ang Thong | 0.554689 |
| 150409 | Khok Phutsa | Ang Thong | 0.445382 |
| 150411 | Bo Rae | Ang Thong | 0.554681 |
| 150412 | Thang Phra | Ang Thong | 0.487728 |
| 150413 | Sam Ngam | Ang Thong | 0.56197 |
| 150414 | Bang Chao Cha | Ang Thong | 0.53577 |
| 150415 | Kham Yat | Ang Thong | 0.433876 |
| 150507 | Chamlong | Ang Thong | 0.462502 |
| 150601 | Phai Chamsin | Ang Thong | 0.528483 |
| 150602 | San Chao Rong Thong | Ang Thong | 0.480736 |
| 150603 | Phai Dam Phatthana | Ang Thong | 0.406245 |
| 150605 | Tha Chang | Ang Thong | 0.450592 |
| 150611 | Si Roi | Ang Thong | 0.538467 |
| 150612 | Muang Tia | Ang Thong | 0.47452 |
| 150613 | Hua Taphan | Ang Thong | 0.497032 |
| 160112 | Talung | Lop Buri | 0.407028 |
| 160119 | Thai Talat | Lop Buri | 0.523116 |
| 160123 | Pho Tru | Lop Buri | 0.437707 |
| 160501 | Tha Wung | Lop Buri | 0.495941 |
| 160502 | Bang Khu | Lop Buri | 0.464633 |
| 160505 | Bang Nga | Lop Buri | 0.422252 |
| 160506 | Khok Salut | Lop Buri | 0.429714 |
| 160508 | Hua Samrong | Lop Buri | 0.449615 |
| 160509 | Lat Sali | Lop Buri | 0.488956 |
| 160510 | Ban Boek | Lop Buri | 0.400591 |
| 170104 | Muang Mu | Sing Buri | 0.457452 |
| 170106 | Ton Pho | Sing Buri | 0.447422 |
| 170107 | Chaksi | Sing Buri | 0.444592 |
| 170303 | Pho Sangkho | Sing Buri | 0.429 |
| 170304 | Tha Kham | Sing Buri | 0.480324 |
| 170401 | Phra Ngam | Sing Buri | 0.536407 |
| 170402 | Phrom Buri | Sing Buri | 0.529196 |
| 170404 | Ban Mo | Sing Buri | 0.492582 |
| 170405 | Ban Paeng | Sing Buri | 0.513964 |
| 170406 | Hua Pa | Sing Buri | 0.514393 |
| 170407 | Rong Chang | Sing Buri | 0.521739 |
| 170501 | Thon Samo | Sing Buri | 0.516208 |
| 170502 | Pho Prachak | Sing Buri | 0.468043 |
| 170503 | Wihan Khao | Sing Buri | 0.503735 |
| 170504 | Phikun Thong | Sing Buri | 0.524232 |
| 180103 | Tha Chai | Chai Nat | 0.424912 |
| 180106 | Hat Tha Sao | Chai Nat | 0.469014 |
| 180109 | Nang Lue | Chai Nat | 0.421349 |
| 180302 | Makham Thao | Chai Nat | 0.40807 |
| 180303 | Nong Noi | Chai Nat | 0.419842 |
| 180601 | Han Kha | Chai Nat | 0.408327 |
| 180607 | Huai Ngu | Chai Nat | 0.479428 |
| 180611 | Sam Ngam Tha Bot | Chai Nat | 0.461395 |
| 190105 | Dao Rueang | Saraburi | 0.41157 |
| 190106 | Na Chong | Saraburi | 0.445807 |
| 190113 | Taling Chan | Saraburi | 0.553059 |
| 190211 | Song Khon | Saraburi | 0.429427 |
| 190301 | Nong Khae | Saraburi | 0.47383 |
| 190302 | Kum Hak | Saraburi | 0.544677 |
| 190303 | Khotchasit | Saraburi | 0.493516 |
| 190304 | Khok Tum | Saraburi | 0.432878 |
| 190307 | Phai Tam | Saraburi | 0.468263 |
| 190311 | Nong Khai Nam | Saraburi | 0.428747 |
| 190312 | Nong Khaem | Saraburi | 0.431707 |
| 190314 | Nong Chorakhe | Saraburi | 0.456596 |
| 190317 | Nong Pling | Saraburi | 0.500789 |
| 190318 | Nong Rong | Saraburi | 0.550185 |
| 190401 | Nong Mu | Saraburi | 0.512657 |
| 190402 | Ban Lam | Saraburi | 0.421625 |
| 190403 | Khlong Ruea | Saraburi | 0.480117 |
| 190404 | Wihan Daeng | Saraburi | 0.534509 |
| 190405 | Nong Suang | Saraburi | 0.515541 |
| 190501 | Nong Saeng | Saraburi | 0.431019 |
| 190503 | Nong Hua Pho | Saraburi | 0.436219 |
| 190504 | Nong Sida | Saraburi | 0.442783 |
| 190505 | Nong Kop | Saraburi | 0.497498 |
| 190506 | Kai Sao | Saraburi | 0.433856 |
| 190507 | Khok Sa-At | Saraburi | 0.46254 |
| 190508 | Muang Wan | Saraburi | 0.41959 |
| 190509 | Khao Din | Saraburi | 0.423903 |
| 190608 | Ban Khrua | Saraburi | 0.418288 |
| 191001 | Sao Hai | Saraburi | 0.454096 |
| 191002 | Ban Yang | Saraburi | 0.481149 |
| 191004 | Ngio Ngam | Saraburi | 0.455503 |
| 191005 | Sala Ri Thai | Saraburi | 0.51179 |
| 191006 | Ton Tan | Saraburi | 0.440848 |
| 191007 | Tha Chang | Saraburi | 0.446416 |
| 191008 | Phraya Thot | Saraburi | 0.411212 |
| 191009 | Muang Ngam | Saraburi | 0.500371 |
| 191010 | Roeng Rang | Saraburi | 0.459869 |
| 191011 | Mueang Kao | Saraburi | 0.467059 |
| 191012 | Suan Dokmai | Saraburi | 0.454425 |
| 191303 | Phueng Ruang | Saraburi | 0.40656 |
| 200108 | Nong Khang Khok | Chon Buri | 0.423387 |
| 200203 | Map Phai | Chon Buri | 0.526325 |
| 200204 | Nong Samsak | Chon Buri | 0.46489 |
| 200502 | Nong Tamlueng | Chon Buri | 0.444231 |
| 200504 | Nong Kakha | Chon Buri | 0.536982 |
| 200505 | Nong Hong | Chon Buri | 0.546259 |
| 200508 | Na Pradu | Chon Buri | 0.463338 |
| 200510 | Ko Loi | Chon Buri | 0.424195 |
| 200511 | Bang Hak | Chon Buri | 0.426594 |
| 200601 | Phanat Nikhom | Chon Buri | 0.469371 |
| 200603 | Wat Luang | Chon Buri | 0.47745 |
| 200606 | Mon Nang | Chon Buri | 0.556687 |
| 200608 | Wat Bot | Chon Buri | 0.525908 |
| 200609 | Kut Ngong | Chon Buri | 0.500361 |
| 200610 | Hua Thanon | Chon Buri | 0.45767 |
| 200615 | Thung Khwang | Chon Buri | 0.497894 |
| 200618 | Ban Chang | Chon Buri | 0.501295 |
| 200620 | Khok Phlo | Chon Buri | 0.444716 |
| 200621 | Rai Lak Thong | Chon Buri | 0.446841 |
| 200622 | Na Matum | Chon Buri | 0.541343 |
| 200801 | Tha Thewawong | Chon Buri | 0.517567 |
| 201003 | Bo Kwang Thong | Chon Buri | 0.43453 |
| 240103 | Ban Mai | Chachoengsao | 0.432592 |
| 240104 | Khlong Na | Chachoengsao | 0.488741 |
| 240105 | Bang Tin Pet | Chachoengsao | 0.491409 |
| 240108 | Bang Kaeo | Chachoengsao | 0.44374 |
| 240112 | Sothon | Chachoengsao | 0.482159 |
| 240113 | Bang Phra | Chachoengsao | 0.541799 |
| 240114 | Bang Kahai | Chachoengsao | 0.45811 |
| 240201 | Bang Khla | Chachoengsao | 0.508972 |
| 240204 | Bang Suan | Chachoengsao | 0.585676 |
| 240209 | Pak Nam | Chachoengsao | 0.522732 |
| 240210 | Tha Thong Lang | Chachoengsao | 0.448658 |
| 240213 | Samet Tai | Chachoengsao | 0.430408 |
| 240214 | Hua Sai | Chachoengsao | 0.435103 |
| 240307 | Yothaka | Chachoengsao | 0.409801 |
| 240402 | Tha Sa-An | Chachoengsao | 0.451749 |
| 240405 | Bang Phueng | Chachoengsao | 0.442336 |
| 240408 | Nong Chok | Chachoengsao | 0.463771 |
| 240409 | Phimpha | Chachoengsao | 0.455165 |
| 240410 | Tha Kham | Chachoengsao | 0.418324 |
| 240412 | Khao Din | Chachoengsao | 0.498332 |
| 240501 | Ban Pho | Chachoengsao | 0.483673 |
| 240502 | Ko Rai | Chachoengsao | 0.432515 |
| 240503 | Khlong Khut | Chachoengsao | 0.454684 |
| 240504 | Khlong Ban Pho | Chachoengsao | 0.539699 |
| 240505 | Khlong Prawet | Chachoengsao | 0.498322 |
| 240506 | Don Sai | Chachoengsao | 0.458388 |
| 240507 | Theppharat | Chachoengsao | 0.465119 |
| 240508 | Tha Phlap | Chachoengsao | 0.524459 |
| 240509 | Nong Tin Nok | Chachoengsao | 0.414356 |
| 240510 | Nong Bua | Chachoengsao | 0.455468 |
| 240511 | Bang Son | Chachoengsao | 0.491226 |
| 240512 | Bang Krut | Chachoengsao | 0.559236 |
| 240514 | Lat Khwang | Chachoengsao | 0.495569 |
| 240515 | Sanam Chan | Chachoengsao | 0.487247 |
| 240516 | Saen Phu Dat | Chachoengsao | 0.4603 |
| 240701 | Bang Kha | Chachoengsao | 0.597878 |
| 240702 | Mueang Mai | Chachoengsao | 0.427945 |
| 241101 | Kon Kaeo | Chachoengsao | 0.429705 |
| 241102 | Khlong Khuean | Chachoengsao | 0.501973 |
| 241105 | Bang Talat | Chachoengsao | 0.574942 |
| 250102 | Rop Mueang | Prachin Buri | 0.453486 |
| 250103 | Wat Bot | Prachin Buri | 0.438129 |
| 250104 | Bang Decha | Prachin Buri | 0.422164 |
| 250105 | Tha Ngam | Prachin Buri | 0.407421 |
| 250107 | Dong Phraram | Prachin Buri | 0.422711 |
| 250108 | Ban Phra | Prachin Buri | 0.40435 |
| 250601 | Ban Sang | Prachin Buri | 0.41858 |
| 250602 | Bang Krabao | Prachin Buri | 0.41766 |
| 250603 | Bang Toei | Prachin Buri | 0.432124 |
| 250604 | Bang Yang | Prachin Buri | 0.434286 |
| 250605 | Bang Taen | Prachin Buri | 0.410193 |
| 250608 | Bang Kham | Prachin Buri | 0.509025 |
| 260105 | Tha Sai | Nakhon Nayok | 0.460731 |
| 260301 | Ban Na | Nakhon Nayok | 0.460575 |
| 260302 | Ban Phrao | Nakhon Nayok | 0.411866 |
| 260303 | Ban Phrik | Nakhon Nayok | 0.492777 |
| 260305 | Thong Lang | Nakhon Nayok | 0.479508 |
| 260306 | Bang O | Nakhon Nayok | 0.42503 |
| 260309 | Khao Phoem | Nakhon Nayok | 0.494211 |
| 260310 | Si Ka-Ang | Nakhon Nayok | 0.435844 |
| 260405 | Bang Sombun | Nakhon Nayok | 0.46099 |
| 620406 | Salok Bat | Kamphaeng Phet | 0.584962 |
| 620411 | Pang Makha | Kamphaeng Phet | 0.594666 |
| 670606 | Nong Yang Thoi | Phetchabun | 0.57835 |
| 700111 | Phikun Thong | Ratchaburi | 0.429762 |
| 700401 | Damnoen Saduak | Ratchaburi | 0.436454 |
| 700402 | Prasatsit | Ratchaburi | 0.503702 |
| 700403 | Si Surat | Ratchaburi | 0.538279 |
| 700404 | Ta Luang | Ratchaburi | 0.501991 |
| 700410 | Si Muen | Ratchaburi | 0.470549 |
| 700411 | Tha Nat | Ratchaburi | 0.412613 |
| 700412 | Khun Phithak | Ratchaburi | 0.472517 |
| 700413 | Don Phi | Ratchaburi | 0.501548 |
| 700604 | Wat Kaeo | Ratchaburi | 0.422371 |
| 700705 | Khlong Ta Kot | Ratchaburi | 0.454715 |
| 700711 | Chamrae | Ratchaburi | 0.409431 |
| 700712 | Soi Fa | Ratchaburi | 0.505517 |
| 720102 | Rua Yai | Suphan Buri | 0.436388 |
| 720103 | Thap Ti Lek | Suphan Buri | 0.450641 |
| 720104 | Tha Rahat | Suphan Buri | 0.493695 |
| 720105 | Phai Khwang | Suphan Buri | 0.431701 |
| 720108 | Don Masang | Suphan Buri | 0.458487 |
| 720109 | Phihan Daeng | Suphan Buri | 0.562507 |
| 720112 | Ban Pho | Suphan Buri | 0.465611 |
| 720113 | Sa Kaeo | Suphan Buri | 0.440962 |
| 720115 | Bang Kung | Suphan Buri | 0.452785 |
| 720118 | Sanam Chai | Suphan Buri | 0.498432 |
| 720119 | Pho Phraya | Suphan Buri | 0.503469 |
| 720120 | Sanam Khli | Suphan Buri | 0.408287 |
| 720402 | Bang Pla Ma | Suphan Buri | 0.42062 |
| 720403 | Takha | Suphan Buri | 0.426461 |
| 720404 | Bang Yai | Suphan Buri | 0.432946 |
| 720405 | Kritsana | Suphan Buri | 0.424546 |
| 720406 | Sali | Suphan Buri | 0.42137 |
| 720410 | Ban Laem | Suphan Buri | 0.490144 |
| 720501 | Si Prachan | Suphan Buri | 0.435236 |
| 720502 | Ban Krang | Suphan Buri | 0.482677 |
| 720503 | Mot Daeng | Suphan Buri | 0.52418 |
| 720504 | Bang Ngam | Suphan Buri | 0.474372 |
| 720507 | Wang Wa | Suphan Buri | 0.456001 |
| 720508 | Wang Nam Sap | Suphan Buri | 0.456861 |
| 720509 | Wang Yang | Suphan Buri | 0.468558 |
| 720704 | Bang Takhian | Suphan Buri | 0.423715 |
| 720705 | Ban Kum | Suphan Buri | 0.449374 |
| 730501 | Bang Len | Nakhon Pathom | 0.430845 |
| 730502 | Bang Pla | Nakhon Pathom | 0.412924 |
| 730504 | Bang Phasi | Nakhon Pathom | 0.435412 |
| 730506 | Bang Sai Pa | Nakhon Pathom | 0.434429 |
| 730507 | Hin Mun | Nakhon Pathom | 0.427326 |
| 730508 | Sai Ngam | Nakhon Pathom | 0.428065 |
| 730509 | Don Tum | Nakhon Pathom | 0.484824 |
| 730512 | Khlong Nok Krathung | Nakhon Pathom | 0.45504 |
| 730514 | Lam Phaya | Nakhon Pathom | 0.447982 |
| 730606 | Sam Phran | Nakhon Pathom | 0.50597 |
| 730615 | Ban Mai | Nakhon Pathom | 0.492812 |
| 740105 | Bang Thorat | Samut Sakhon | 0.439584 |
| 740106 | Kalong | Samut Sakhon | 0.419155 |
| 740107 | Na Khok | Samut Sakhon | 0.425423 |
| 740302 | Lak Sam | Samut Sakhon | 0.440132 |
| 740303 | Yok Krabat | Samut Sakhon | 0.481284 |
| 740304 | Rong Khe | Samut Sakhon | 0.506671 |
| 740305 | Nong Song Hong | Samut Sakhon | 0.471652 |
| 740306 | Nong Bua | Samut Sakhon | 0.447162 |
| 750103 | Lat Yai | Samut Songkhram | 0.485458 |
| 750104 | Ban Prok | Samut Songkhram | 0.485523 |
| 750105 | Bang Kaeo | Samut Songkhram | 0.402518 |
| 750106 | Thai Hat | Samut Songkhram | 0.423432 |
| 750108 | Khlong Khoen | Samut Songkhram | 0.535794 |
| 750110 | Nang Takhian | Samut Songkhram | 0.545122 |
| 750201 | Kradang Nga | Samut Songkhram | 0.50895 |
| 750202 | Bang Sakae | Samut Songkhram | 0.473557 |
| 750203 | Bang Yi Rong | Samut Songkhram | 0.457617 |
| 750204 | Rong Hip | Samut Songkhram | 0.499626 |
| 750205 | Bang Khonthi | Samut Songkhram | 0.542088 |
| 750206 | Don Manora | Samut Songkhram | 0.494316 |
| 750207 | Bang Phrom | Samut Songkhram | 0.495772 |
| 750208 | Bang Kung | Samut Songkhram | 0.517038 |
| 750209 | Chom Pluak | Samut Songkhram | 0.459021 |
| 750210 | Bang Nok Khwaek | Samut Songkhram | 0.512629 |
| 750211 | Yai Phaeng | Samut Songkhram | 0.49368 |
| 750212 | Bang Krabue | Samut Songkhram | 0.471663 |
| 750213 | Ban Pramot | Samut Songkhram | 0.48813 |
| 750301 | Amphawa | Samut Songkhram | 0.489902 |
| 750302 | Suan Luang | Samut Songkhram | 0.473267 |
| 750303 | Tha Kha | Samut Songkhram | 0.52575 |
| 750305 | Mueang Mai | Samut Songkhram | 0.41888 |
| 750306 | Bang Chang | Samut Songkhram | 0.495864 |
| 750307 | Khwae Om | Samut Songkhram | 0.494244 |

**Table S3** The list of low risk subdistricts for NiV occurrence at subdistrict level

| **Geocode** | **Name of the subdistrict** | **Province** | **Probability of NiV occurrence** |
| --- | --- | --- | --- |
| 100101 | Phraborom Maharatchawang | Bangkok | 0.203767 |
| 100102 | Wang Burapha Phirom | Bangkok | 0.215074 |
| 100103 | Wat Ratchabophit | Bangkok | 0.209058 |
| 100104 | Samran Rat | Bangkok | 0.215872 |
| 100106 | Sao Chingcha | Bangkok | 0.2031 |
| 100109 | Chana Songkhram | Bangkok | 0.205733 |
| 100112 | Wat Sam Phraya | Bangkok | 0.215295 |
| 100202 | Wachira Phayaban | Bangkok | 0.224554 |
| 100204 | Si Yaek Mahanak | Bangkok | 0.217475 |
| 100401 | Maha Phruettharam | Bangkok | 0.248325 |
| 100402 | Si Lom | Bangkok | 0.233395 |
| 100403 | Suriyawong | Bangkok | 0.200797 |
| 100404 | Bang Rak | Bangkok | 0.320776 |
| 100405 | Si Phraya | Bangkok | 0.220706 |
| 100508 | Tha Raeng | Bangkok | 0.258836 |
| 100701 | Rong Mueang | Bangkok | 0.205227 |
| 100801 | Pom Prap | Bangkok | 0.220736 |
| 100802 | Wat Thepsirin | Bangkok | 0.217723 |
| 100803 | Khlong Mahanak | Bangkok | 0.222832 |
| 100804 | Ban Bat | Bangkok | 0.227188 |
| 100805 | Wat Sommanat | Bangkok | 0.204542 |
| 100905 | Bang Chak | Bangkok | 0.289607 |
| 101001 | Min Buri | Bangkok | 0.33447 |
| 101102 | Khlong Song Ton Nun | Bangkok | 0.203912 |
| 101103 | Khlong Sam Prawet | Bangkok | 0.211436 |
| 101104 | Lam Pla Thio | Bangkok | 0.355786 |
| 101105 | Thap Yao | Bangkok | 0.277262 |
| 101106 | Khum Thong | Bangkok | 0.304776 |
| 101203 | Chong Nonsi | Bangkok | 0.290126 |
| 101204 | Bang Phongphang | Bangkok | 0.328238 |
| 101301 | Chakkrawat | Bangkok | 0.257489 |
| 101302 | Samphanthawong | Bangkok | 0.242352 |
| 101303 | Talat Noi | Bangkok | 0.288445 |
| 101501 | Wat Kanlaya | Bangkok | 0.247776 |
| 101502 | Hiranruchi | Bangkok | 0.245915 |
| 101503 | Bang Yi Ruea | Bangkok | 0.234422 |
| 101504 | Bukkhalo | Bangkok | 0.22259 |
| 101505 | Talat Phlu | Bangkok | 0.244209 |
| 101506 | Dao Khanong | Bangkok | 0.251285 |
| 101507 | Samre | Bangkok | 0.279698 |
| 101601 | Wat Arun | Bangkok | 0.235628 |
| 101702 | Bang Kapi | Bangkok | 0.213475 |
| 101802 | Khlong San | Bangkok | 0.278197 |
| 101803 | Bang Lamphu Lang | Bangkok | 0.242179 |
| 101804 | Khlong Ton Sai | Bangkok | 0.216996 |
| 101901 | Khlong Chak Phra | Bangkok | 0.232606 |
| 101904 | Bang Phrom | Bangkok | 0.264344 |
| 101905 | Bang Ramat | Bangkok | 0.234917 |
| 101907 | Bang Chueak Nang | Bangkok | 0.236334 |
| 102105 | Tha Kham | Bangkok | 0.32065 |
| 102107 | Samae Dam | Bangkok | 0.242509 |
| 102201 | Bang Wa | Bangkok | 0.218504 |
| 102206 | Bang Chak | Bangkok | 0.283888 |
| 102207 | Bang Waek | Bangkok | 0.266867 |
| 102208 | Khlong Khwang | Bangkok | 0.23369 |
| 102209 | Pak Khlong Phasi Charoen | Bangkok | 0.242637 |
| 102210 | Khuha Sawan | Bangkok | 0.298148 |
| 102401 | Rat Burana | Bangkok | 0.329983 |
| 102402 | Bang Pakok | Bangkok | 0.339493 |
| 102801 | Thung Wat Don | Bangkok | 0.294862 |
| 102802 | Yan Nawa | Bangkok | 0.230161 |
| 102803 | Thung Mahamek | Bangkok | 0.218293 |
| 103101 | Bang Kho Laem | Bangkok | 0.316873 |
| 103102 | Wat Phraya Krai | Bangkok | 0.291047 |
| 103103 | Bang Khlo | Bangkok | 0.294163 |
| 103201 | Prawet | Bangkok | 0.269187 |
| 103202 | Nong Bon | Bangkok | 0.282591 |
| 103203 | Dokmai | Bangkok | 0.330648 |
| 103301 | Khlong Toei | Bangkok | 0.25579 |
| 103302 | Khlong Tan | Bangkok | 0.226323 |
| 103303 | Phra Khanong | Bangkok | 0.291748 |
| 103401 | Suan Luang | Bangkok | 0.238039 |
| 103501 | Bang Khun Thian | Bangkok | 0.253661 |
| 103502 | Bang Kho | Bangkok | 0.262656 |
| 103503 | Bang Mot | Bangkok | 0.279205 |
| 103504 | Chom Thong | Bangkok | 0.270255 |
| 103901 | Khlong Toei Nuea | Bangkok | 0.203653 |
| 103902 | Khlong Tan Nuea | Bangkok | 0.22345 |
| 103903 | Phra Khanong Nuea | Bangkok | 0.268969 |
| 104201 | Sai Mai | Bangkok | 0.248935 |
| 104202 | O Ngoen | Bangkok | 0.357873 |
| 104301 | Khan Na Yao | Bangkok | 0.258679 |
| 104401 | Saphan Sung | Bangkok | 0.268875 |
| 104601 | Sam Wa Tawan Tok | Bangkok | 0.399884 |
| 104603 | Bang Chan | Bangkok | 0.369341 |
| 104701 | Bang Na | Bangkok | 0.301932 |
| 104901 | Bang Mot | Bangkok | 0.344489 |
| 110101 | Pak Nam | Samut Prakan | 0.392952 |
| 110102 | Samrong Nuea | Samut Prakan | 0.315184 |
| 110103 | Bang Mueang | Samut Prakan | 0.340361 |
| 110108 | Bang Pu Mai | Samut Prakan | 0.317574 |
| 110110 | Phraeksa | Samut Prakan | 0.332173 |
| 110112 | Bang Pu | Samut Prakan | 0.279882 |
| 110114 | Bang Mueang Mai | Samut Prakan | 0.308155 |
| 110115 | Thepharak | Samut Prakan | 0.336917 |
| 110116 | Thai Ban Mai | Samut Prakan | 0.349406 |
| 110117 | Phraeksa Mai | Samut Prakan | 0.319802 |
| 110201 | Bang Bo | Samut Prakan | 0.283165 |
| 110202 | Ban Rakat | Samut Prakan | 0.344784 |
| 110203 | Bang Phli Noi | Samut Prakan | 0.379845 |
| 110204 | Bang Phriang | Samut Prakan | 0.234937 |
| 110205 | Khlong Dan | Samut Prakan | 0.241712 |
| 110206 | Khlong Suan | Samut Prakan | 0.389825 |
| 110207 | Preng | Samut Prakan | 0.30637 |
| 110301 | Bang Phli Yai | Samut Prakan | 0.325773 |
| 110302 | Bang Kaeo | Samut Prakan | 0.34926 |
| 110303 | Bang Pla | Samut Prakan | 0.297605 |
| 110304 | Bang Chalong | Samut Prakan | 0.227193 |
| 110308 | Racha Thewa | Samut Prakan | 0.203063 |
| 110402 | Bang Phueng | Samut Prakan | 0.399183 |
| 110403 | Bang Chak | Samut Prakan | 0.378558 |
| 110404 | Bang Khru | Samut Prakan | 0.386297 |
| 110407 | Samrong Tai | Samut Prakan | 0.328138 |
| 110408 | Bang Yo | Samut Prakan | 0.372813 |
| 110409 | Bang Kachao | Samut Prakan | 0.351984 |
| 110410 | Bang Nam Phueng | Samut Prakan | 0.391878 |
| 110412 | Bang Ko Bua | Samut Prakan | 0.381697 |
| 110414 | Samrong | Samut Prakan | 0.359887 |
| 110415 | Samrong Klang | Samut Prakan | 0.380451 |
| 110601 | Bang Sao Thong | Samut Prakan | 0.230745 |
| 110602 | Sisa Chorakhe Noi | Samut Prakan | 0.239322 |
| 110603 | Sisa Chorakhe Yai | Samut Prakan | 0.22013 |
| 120101 | Suan Yai | Nonthaburi | 0.234437 |
| 120106 | Bang Phai | Nonthaburi | 0.241603 |
| 120107 | Bang Si Mueang | Nonthaburi | 0.222896 |
| 120108 | Bang Krang | Nonthaburi | 0.286615 |
| 120110 | Bang Rak Noi | Nonthaburi | 0.220492 |
| 120201 | Wat Chalo | Nonthaburi | 0.260866 |
| 120203 | Bang Si Thong | Nonthaburi | 0.223257 |
| 120204 | Bang Khanun | Nonthaburi | 0.27469 |
| 120205 | Bang Khun Kong | Nonthaburi | 0.284374 |
| 120206 | Bang Khu Wiang | Nonthaburi | 0.23627 |
| 120207 | Mahasawat | Nonthaburi | 0.252229 |
| 120208 | Plai Bang | Nonthaburi | 0.2433 |
| 120209 | Sala Klang | Nonthaburi | 0.209207 |
| 120301 | Bang Muang | Nonthaburi | 0.270705 |
| 120303 | Bang Len | Nonthaburi | 0.277407 |
| 120305 | Bang Yai | Nonthaburi | 0.232925 |
| 120402 | Bang Bua Thong | Nonthaburi | 0.236514 |
| 120403 | Bang Rak Yai | Nonthaburi | 0.247069 |
| 120404 | Bang Khu Rat | Nonthaburi | 0.216947 |
| 120405 | Lahan | Nonthaburi | 0.208548 |
| 120406 | Lam Pho | Nonthaburi | 0.213961 |
| 120501 | Sai Noi | Nonthaburi | 0.258328 |
| 120502 | Rat Niyom | Nonthaburi | 0.369217 |
| 120503 | Nong Phrao Ngai | Nonthaburi | 0.296551 |
| 120504 | Sai Yai | Nonthaburi | 0.356931 |
| 120506 | Khlong Khwang | Nonthaburi | 0.363225 |
| 120507 | Thawi Watthana | Nonthaburi | 0.318894 |
| 120605 | Bang Tanai | Nonthaburi | 0.279877 |
| 120606 | Khlong Phra Udom | Nonthaburi | 0.311155 |
| 120607 | Tha It | Nonthaburi | 0.260233 |
| 120608 | Ko Kret | Nonthaburi | 0.290355 |
| 120609 | Om Kret | Nonthaburi | 0.28845 |
| 120610 | Khlong Khoi | Nonthaburi | 0.303484 |
| 120611 | Bang Phlap | Nonthaburi | 0.203963 |
| 130101 | Bang Prok | Pathum Thani | 0.34456 |
| 130103 | Ban Klang | Pathum Thani | 0.279174 |
| 130104 | Ban Chang | Pathum Thani | 0.386048 |
| 130105 | Ban Krachaeng | Pathum Thani | 0.381272 |
| 130106 | Bang Khayaeng | Pathum Thani | 0.214012 |
| 130107 | Bang Khu Wat | Pathum Thani | 0.202738 |
| 130108 | Bang Luang | Pathum Thani | 0.370613 |
| 130109 | Bang Duea | Pathum Thani | 0.337111 |
| 130110 | Bang Phut | Pathum Thani | 0.317123 |
| 130111 | Bang Phun | Pathum Thani | 0.229681 |
| 130112 | Bang Kadi | Pathum Thani | 0.262796 |
| 130113 | Suan Phrik Thai | Pathum Thani | 0.302946 |
| 130201 | Khlong Nueng | Pathum Thani | 0.244045 |
| 130202 | Khlong Song | Pathum Thani | 0.291937 |
| 130203 | Khlong Sam | Pathum Thani | 0.24285 |
| 130204 | Khlong Si | Pathum Thani | 0.273791 |
| 130205 | Khlong Ha | Pathum Thani | 0.285333 |
| 130206 | Khlong Hok | Pathum Thani | 0.27826 |
| 130207 | Khlong Chet | Pathum Thani | 0.285561 |
| 130301 | Pracha Thipat | Pathum Thani | 0.208449 |
| 130302 | Bueng Yi Tho | Pathum Thani | 0.287384 |
| 130303 | Rangsit | Pathum Thani | 0.337722 |
| 130304 | Lam Phak Kut | Pathum Thani | 0.299141 |
| 130305 | Bueng Sanan | Pathum Thani | 0.386764 |
| 130306 | Bueng Nam Rak | Pathum Thani | 0.29525 |
| 130401 | Bueng Ba | Pathum Thani | 0.315118 |
| 130402 | Bueng Bon | Pathum Thani | 0.292852 |
| 130404 | Bueng Cham O | Pathum Thani | 0.334089 |
| 130405 | Nong Sam Wang | Pathum Thani | 0.296707 |
| 130501 | Rahaeng | Pathum Thani | 0.381434 |
| 130502 | Lat Lum Kaeo | Pathum Thani | 0.335806 |
| 130505 | Khlong Phra Udom | Pathum Thani | 0.30782 |
| 130507 | Na Mai | Pathum Thani | 0.340407 |
| 130602 | Lat Sawai | Pathum Thani | 0.270798 |
| 130603 | Bueng Kham Phoi | Pathum Thani | 0.392478 |
| 130607 | Bueng Kho Hai | Pathum Thani | 0.373233 |
| 130706 | Chiang Rak Yai | Pathum Thani | 0.356889 |
| 140101 | Pratu Chai | Phra Nakhon Si Ayutthaya | 0.372622 |
| 140106 | Phai Ling | Phra Nakhon Si Ayutthaya | 0.363887 |
| 140107 | Pak Kran | Phra Nakhon Si Ayutthaya | 0.398368 |
| 140108 | Phu Khao Thong | Phra Nakhon Si Ayutthaya | 0.389472 |
| 140112 | Wat Tum | Phra Nakhon Si Ayutthaya | 0.383428 |
| 140114 | Lumphli | Phra Nakhon Si Ayutthaya | 0.348142 |
| 140201 | Tha Ruea | Phra Nakhon Si Ayutthaya | 0.326354 |
| 140204 | Ban Rom | Phra Nakhon Si Ayutthaya | 0.331233 |
| 140206 | Wang Daeng | Phra Nakhon Si Ayutthaya | 0.376755 |
| 140208 | Pak Tha | Phra Nakhon Si Ayutthaya | 0.366132 |
| 140304 | Ban Chung | Phra Nakhon Si Ayutthaya | 0.394441 |
| 140309 | Nong Pling | Phra Nakhon Si Ayutthaya | 0.387899 |
| 140405 | Na Mai | Phra Nakhon Si Ayutthaya | 0.390429 |
| 140406 | Bang Yi Tho | Phra Nakhon Si Ayutthaya | 0.321734 |
| 140409 | Chang Lek | Phra Nakhon Si Ayutthaya | 0.315046 |
| 140411 | Ban Klueng | Phra Nakhon Si Ayutthaya | 0.370101 |
| 140412 | Chang Noi | Phra Nakhon Si Ayutthaya | 0.391627 |
| 140414 | Phai Phra | Phra Nakhon Si Ayutthaya | 0.336693 |
| 140418 | Ban Ko | Phra Nakhon Si Ayutthaya | 0.394857 |
| 140422 | Chiang Rak Noi | Phra Nakhon Si Ayutthaya | 0.399711 |
| 140501 | Bang Ban | Phra Nakhon Si Ayutthaya | 0.358454 |
| 140502 | Wat Yom | Phra Nakhon Si Ayutthaya | 0.314069 |
| 140504 | Saphan Thai | Phra Nakhon Si Ayutthaya | 0.313746 |
| 140505 | Mahaphram | Phra Nakhon Si Ayutthaya | 0.395049 |
| 140506 | Kop Chao | Phra Nakhon Si Ayutthaya | 0.247934 |
| 140507 | Ban Khlang | Phra Nakhon Si Ayutthaya | 0.306095 |
| 140508 | Phra Khao | Phra Nakhon Si Ayutthaya | 0.330553 |
| 140509 | Namtao | Phra Nakhon Si Ayutthaya | 0.30902 |
| 140510 | Thang Chang | Phra Nakhon Si Ayutthaya | 0.296886 |
| 140511 | Wat Taku | Phra Nakhon Si Ayutthaya | 0.290549 |
| 140512 | Bang Luang | Phra Nakhon Si Ayutthaya | 0.261376 |
| 140513 | Bang Luang Dot | Phra Nakhon Si Ayutthaya | 0.30012 |
| 140514 | Bang Hak | Phra Nakhon Si Ayutthaya | 0.347815 |
| 140516 | Ban Kum | Phra Nakhon Si Ayutthaya | 0.389537 |
| 140607 | Ban Wa | Phra Nakhon Si Ayutthaya | 0.396171 |
| 140710 | Thap Nam | Phra Nakhon Si Ayutthaya | 0.388514 |
| 140717 | Ban Khlo | Phra Nakhon Si Ayutthaya | 0.393762 |
| 140801 | Phak Hai | Phra Nakhon Si Ayutthaya | 0.21828 |
| 140802 | Ammarit | Phra Nakhon Si Ayutthaya | 0.282784 |
| 140803 | Ban Khae | Phra Nakhon Si Ayutthaya | 0.270231 |
| 140804 | Lat Nam Khem | Phra Nakhon Si Ayutthaya | 0.295812 |
| 140805 | Ta Lan | Phra Nakhon Si Ayutthaya | 0.23493 |
| 140806 | Tha Din Daeng | Phra Nakhon Si Ayutthaya | 0.212947 |
| 140808 | Na Khu | Phra Nakhon Si Ayutthaya | 0.246522 |
| 140809 | Kudi | Phra Nakhon Si Ayutthaya | 0.241172 |
| 140811 | Khok Chang | Phra Nakhon Si Ayutthaya | 0.302059 |
| 140815 | Na Khok | Phra Nakhon Si Ayutthaya | 0.272539 |
| 140816 | Ban Yai | Phra Nakhon Si Ayutthaya | 0.251951 |
| 140901 | Phachi | Phra Nakhon Si Ayutthaya | 0.31144 |
| 140902 | Khok Muang | Phra Nakhon Si Ayutthaya | 0.344208 |
| 140903 | Rasom | Phra Nakhon Si Ayutthaya | 0.369794 |
| 140906 | Phai Lom | Phra Nakhon Si Ayutthaya | 0.362895 |
| 140907 | Krachio | Phra Nakhon Si Ayutthaya | 0.291019 |
| 141001 | Lat Bua Luang | Phra Nakhon Si Ayutthaya | 0.241222 |
| 141002 | Lak Chai | Phra Nakhon Si Ayutthaya | 0.316543 |
| 141003 | Sam Mueang | Phra Nakhon Si Ayutthaya | 0.346475 |
| 141006 | Khu Salot | Phra Nakhon Si Ayutthaya | 0.306951 |
| 141101 | Lam Ta Sao | Phra Nakhon Si Ayutthaya | 0.381667 |
| 141103 | Wang Noi | Phra Nakhon Si Ayutthaya | 0.364909 |
| 141104 | Lam Sai | Phra Nakhon Si Ayutthaya | 0.396514 |
| 141107 | Han Taphao | Phra Nakhon Si Ayutthaya | 0.346996 |
| 141108 | Wang Chula | Phra Nakhon Si Ayutthaya | 0.359194 |
| 141109 | Khao Ngam | Phra Nakhon Si Ayutthaya | 0.38308 |
| 141110 | Chamaep | Phra Nakhon Si Ayutthaya | 0.352362 |
| 141201 | Sena | Phra Nakhon Si Ayutthaya | 0.206118 |
| 141202 | Ban Phaen | Phra Nakhon Si Ayutthaya | 0.269882 |
| 141203 | Chao Chet | Phra Nakhon Si Ayutthaya | 0.21591 |
| 141204 | Sam Ko | Phra Nakhon Si Ayutthaya | 0.212671 |
| 141205 | Bang Nom Kho | Phra Nakhon Si Ayutthaya | 0.277017 |
| 141206 | Hua Wiang | Phra Nakhon Si Ayutthaya | 0.223746 |
| 141207 | Man Wichai | Phra Nakhon Si Ayutthaya | 0.225613 |
| 141208 | Ban Pho | Phra Nakhon Si Ayutthaya | 0.240793 |
| 141210 | Ban Krathum | Phra Nakhon Si Ayutthaya | 0.228337 |
| 141211 | Ban Thaeo | Phra Nakhon Si Ayutthaya | 0.211771 |
| 141213 | Sam Tum | Phra Nakhon Si Ayutthaya | 0.27717 |
| 141216 | Ban Luang | Phra Nakhon Si Ayutthaya | 0.242483 |
| 141301 | Bang Sai | Phra Nakhon Si Ayutthaya | 0.279545 |
| 141302 | Kaeo Fa | Phra Nakhon Si Ayutthaya | 0.29252 |
| 141303 | Tao Lao | Phra Nakhon Si Ayutthaya | 0.243741 |
| 141305 | Thep Mongkhon | Phra Nakhon Si Ayutthaya | 0.333285 |
| 141306 | Wang Phatthana | Phra Nakhon Si Ayutthaya | 0.308009 |
| 141403 | Sam Bandit | Phra Nakhon Si Ayutthaya | 0.390323 |
| 141405 | Nong Mai Sung | Phra Nakhon Si Ayutthaya | 0.292449 |
| 141407 | Sena | Phra Nakhon Si Ayutthaya | 0.300646 |
| 141408 | Nong Nam Som | Phra Nakhon Si Ayutthaya | 0.382197 |
| 141409 | Pho Sao Han | Phra Nakhon Si Ayutthaya | 0.37723 |
| 141505 | Bang Na | Phra Nakhon Si Ayutthaya | 0.381491 |
| 141509 | Ban Na | Phra Nakhon Si Ayutthaya | 0.387064 |
| 141605 | Song Hong | Phra Nakhon Si Ayutthaya | 0.30973 |
| 150101 | Talat Luang | Ang Thong | 0.367535 |
| 150107 | Mahatthai | Ang Thong | 0.384605 |
| 150209 | Tri Narong | Ang Thong | 0.39009 |
| 150302 | Pa Mok | Ang Thong | 0.383271 |
| 150305 | Bang Sadet | Ang Thong | 0.397239 |
| 150307 | Ekkarat | Ang Thong | 0.351207 |
| 150308 | Phong Pheng | Ang Thong | 0.328888 |
| 150405 | Ram Masak | Ang Thong | 0.330979 |
| 150410 | Yang Chai | Ang Thong | 0.396946 |
| 150501 | Sawaeng Ha | Ang Thong | 0.32085 |
| 150502 | Si Phran | Ang Thong | 0.380574 |
| 150503 | Ban Phran | Ang Thong | 0.358217 |
| 150504 | Wang Nam Yen | Ang Thong | 0.277692 |
| 150505 | Si Bua Thong | Ang Thong | 0.241572 |
| 150506 | Huai Phai | Ang Thong | 0.335172 |
| 150604 | Sao Rong Hai | Ang Thong | 0.377579 |
| 150606 | Yi Lon | Ang Thong | 0.375757 |
| 150607 | Bang Chak | Ang Thong | 0.315782 |
| 150608 | Huai Khan Laen | Ang Thong | 0.328296 |
| 150609 | Khlong Khanak | Ang Thong | 0.315759 |
| 150610 | Phai Wong | Ang Thong | 0.341042 |
| 150614 | Lak Kaeo | Ang Thong | 0.255815 |
| 150615 | Talat Mai | Ang Thong | 0.282699 |
| 150701 | Sam Ko | Ang Thong | 0.329494 |
| 150702 | Ratsadon Phatthana | Ang Thong | 0.353009 |
| 150703 | Optom | Ang Thong | 0.384033 |
| 150704 | Pho Muang Phan | Ang Thong | 0.34548 |
| 150705 | Mongkhontham Nimit | Ang Thong | 0.33408 |
| 160104 | Kong Thanu | Lop Buri | 0.38025 |
| 160107 | Khok Kathiam | Lop Buri | 0.220692 |
| 160109 | Khok Tum | Lop Buri | 0.247182 |
| 160110 | Ngio Rai | Lop Buri | 0.369663 |
| 160111 | Don Pho | Lop Buri | 0.218916 |
| 160117 | Bang Khan Mak | Lop Buri | 0.317713 |
| 160118 | Ban Khoi | Lop Buri | 0.354117 |
| 160121 | Phrommat | Lop Buri | 0.279701 |
| 160122 | Pho Kao Ton | Lop Buri | 0.374219 |
| 160124 | Si Khlong | Lop Buri | 0.349196 |
| 160203 | Manao Wan | Lop Buri | 0.368909 |
| 160205 | Khok Salung | Lop Buri | 0.204967 |
| 160207 | Nong Bua | Lop Buri | 0.229086 |
| 160308 | Phaniat | Lop Buri | 0.200177 |
| 160407 | Tha Din Dam | Lop Buri | 0.20936 |
| 160414 | Tha Manao | Lop Buri | 0.377047 |
| 160503 | Pho Talat Kaeo | Lop Buri | 0.376467 |
| 160504 | Bang Li | Lop Buri | 0.296459 |
| 160507 | Khao Samo Khon | Lop Buri | 0.315292 |
| 160511 | Mutchalin | Lop Buri | 0.379397 |
| 160605 | Ban Chi | Lop Buri | 0.268665 |
| 160613 | Bang Kham | Lop Buri | 0.300425 |
| 160615 | Chon Muang | Lop Buri | 0.268088 |
| 170101 | Bang Phutsa | Sing Buri | 0.326942 |
| 170102 | Bang Man | Sing Buri | 0.2894 |
| 170103 | Phok Ruam | Sing Buri | 0.253509 |
| 170108 | Bang Krabue | Sing Buri | 0.300287 |
| 170201 | Sing | Sing Buri | 0.314807 |
| 170202 | Mai Dat | Sing Buri | 0.319002 |
| 170203 | Choeng Klat | Sing Buri | 0.267403 |
| 170204 | Pho Chon Kai | Sing Buri | 0.232181 |
| 170206 | Ban Cha | Sing Buri | 0.221356 |
| 170302 | Bang Rachan | Sing Buri | 0.322495 |
| 170305 | Kho Sai | Sing Buri | 0.264936 |
| 170603 | Thap Ya | Sing Buri | 0.207106 |
| 170606 | Tha Ngam | Sing Buri | 0.321119 |
| 170607 | Namtan | Sing Buri | 0.229233 |
| 180101 | Nai Mueang | Chai Nat | 0.324384 |
| 180102 | Ban Kluai | Chai Nat | 0.293927 |
| 180104 | Chai Nat | Chai Nat | 0.389264 |
| 180105 | Khao Tha Phra | Chai Nat | 0.278009 |
| 180107 | Thammamun | Chai Nat | 0.279773 |
| 180201 | Khung Samphao | Chai Nat | 0.229779 |
| 180301 | Wat Sing | Chai Nat | 0.308588 |
| 180304 | Nong Bua | Chai Nat | 0.251715 |
| 180306 | Nong Khun | Chai Nat | 0.264633 |
| 180311 | Wang Man | Chai Nat | 0.292247 |
| 180401 | Sapphaya | Chai Nat | 0.209615 |
| 180404 | Pho Nang Dam Tok | Chai Nat | 0.219742 |
| 180405 | Pho Nang Dam Ok | Chai Nat | 0.202846 |
| 180406 | Bang Luang | Chai Nat | 0.281398 |
| 180501 | Phraek Si Racha | Chai Nat | 0.35561 |
| 180502 | Thiang Thae | Chai Nat | 0.346615 |
| 180503 | Huai Krot | Chai Nat | 0.250313 |
| 180504 | Pho Ngam | Chai Nat | 0.223042 |
| 180505 | Bang Khut | Chai Nat | 0.209012 |
| 180506 | Dong Khon | Chai Nat | 0.234959 |
| 180507 | Don Kam | Chai Nat | 0.209305 |
| 180602 | Ban Chian | Chai Nat | 0.294678 |
| 180605 | Phrai Nok Yung | Chai Nat | 0.237611 |
| 180606 | Nong Saeng | Chai Nat | 0.398555 |
| 180608 | Wang Kai Thuean | Chai Nat | 0.383394 |
| 180609 | Den Yai | Chai Nat | 0.300428 |
| 190101 | Pak Phriao | Saraburi | 0.332119 |
| 190107 | Khok Sawang | Saraburi | 0.394927 |
| 190108 | Nong No | Saraburi | 0.385045 |
| 190109 | Nong Yao | Saraburi | 0.361528 |
| 190110 | Pak Khao San | Saraburi | 0.351231 |
| 190111 | Nong Pla Lai | Saraburi | 0.300072 |
| 190112 | Kut Nok Plao | Saraburi | 0.27996 |
| 190114 | Takut | Saraburi | 0.397165 |
| 190202 | Thap Kwang | Saraburi | 0.210012 |
| 190203 | Tan Diao | Saraburi | 0.245503 |
| 190204 | Huai Haeng | Saraburi | 0.242911 |
| 190207 | Ban That | Saraburi | 0.229828 |
| 190209 | Tha Tum | Saraburi | 0.211219 |
| 190210 | Cha-Om | Saraburi | 0.27502 |
| 190212 | Tao Pun | Saraburi | 0.335291 |
| 190305 | Khok Yae | Saraburi | 0.396336 |
| 190306 | Bua Loi | Saraburi | 0.383943 |
| 190308 | Phon Thong | Saraburi | 0.368197 |
| 190309 | Huai Khamin | Saraburi | 0.35024 |
| 190310 | Huai Sai | Saraburi | 0.35442 |
| 190313 | Nong Chik | Saraburi | 0.358061 |
| 190315 | Nong Nak | Saraburi | 0.360709 |
| 190316 | Nong Pla Mo | Saraburi | 0.397595 |
| 190406 | Charoen Tham | Saraburi | 0.375107 |
| 190601 | Ban Mo | Saraburi | 0.270106 |
| 190602 | Bang Khamot | Saraburi | 0.385514 |
| 190603 | Sang Sok | Saraburi | 0.325992 |
| 190604 | Talat Noi | Saraburi | 0.245897 |
| 190605 | Horathep | Saraburi | 0.255009 |
| 190606 | Khok Yai | Saraburi | 0.268903 |
| 190607 | Phai Khwang | Saraburi | 0.325359 |
| 190609 | Nong Bua | Saraburi | 0.337853 |
| 190701 | Don Phut | Saraburi | 0.363762 |
| 190702 | Phai Lio | Saraburi | 0.294125 |
| 190703 | Ban Luang | Saraburi | 0.355833 |
| 190704 | Dong Ta-Ngao | Saraburi | 0.341252 |
| 190804 | Ban Prong | Saraburi | 0.350703 |
| 190903 | Than Kasem | Saraburi | 0.235441 |
| 190906 | Khao Wong | Saraburi | 0.241364 |
| 190907 | Huai Pa Wai | Saraburi | 0.332558 |
| 190908 | Phu Krang | Saraburi | 0.22071 |
| 191003 | Hua Pluak | Saraburi | 0.357345 |
| 191201 | Salaeng Phan | Saraburi | 0.202056 |
| 191202 | Kham Phran | Saraburi | 0.247285 |
| 191301 | Khao Din Phatthana | Saraburi | 0.311212 |
| 191302 | Ban Kaeng | Saraburi | 0.375965 |
| 191304 | Phu Khae | Saraburi | 0.245102 |
| 191305 | Huai Bong | Saraburi | 0.375751 |
| 200101 | Bang Pla Soi | Chon Buri | 0.341284 |
| 200102 | Makham Yong | Chon Buri | 0.333083 |
| 200103 | Ban Khot | Chon Buri | 0.336233 |
| 200104 | Saen Suk | Chon Buri | 0.320673 |
| 200105 | Ban Suan | Chon Buri | 0.317388 |
| 200106 | Nong Ri | Chon Buri | 0.362578 |
| 200107 | Na Pa | Chon Buri | 0.282276 |
| 200109 | Don Hua Lo | Chon Buri | 0.235477 |
| 200110 | Nong Mai Daeng | Chon Buri | 0.27764 |
| 200111 | Bang Sai | Chon Buri | 0.290536 |
| 200112 | Khlong Tamru | Chon Buri | 0.29733 |
| 200113 | Mueang | Chon Buri | 0.375614 |
| 200114 | Ban Puek | Chon Buri | 0.322232 |
| 200115 | Huai Kapi | Chon Buri | 0.379223 |
| 200116 | Samet | Chon Buri | 0.362207 |
| 200117 | Ang Sila | Chon Buri | 0.35362 |
| 200118 | Samnak Bok | Chon Buri | 0.341469 |
| 200201 | Ban Bueng | Chon Buri | 0.244977 |
| 200202 | Khlong Kio | Chon Buri | 0.207416 |
| 200205 | Nong Bon Daeng | Chon Buri | 0.294614 |
| 200206 | Nong Chak | Chon Buri | 0.248983 |
| 200207 | Nong I Run | Chon Buri | 0.298112 |
| 200304 | Hang Sung | Chon Buri | 0.222216 |
| 200407 | Takhian Tia | Chon Buri | 0.214091 |
| 200501 | Phan Thong | Chon Buri | 0.375091 |
| 200503 | Map Pong | Chon Buri | 0.379156 |
| 200506 | Khok Khi Non | Chon Buri | 0.376113 |
| 200507 | Ban Kao | Chon Buri | 0.288161 |
| 200509 | Bang Nang | Chon Buri | 0.385096 |
| 200604 | Ban Soet | Chon Buri | 0.399182 |
| 200607 | Sa Si Liam | Chon Buri | 0.390768 |
| 200611 | Tha Kham | Chon Buri | 0.366296 |
| 200613 | Nong Prue | Chon Buri | 0.396818 |
| 200614 | Nong Khayat | Chon Buri | 0.360475 |
| 200616 | Nong Hiang | Chon Buri | 0.359241 |
| 200701 | Si Racha | Chon Buri | 0.216391 |
| 200707 | Bang Phra | Chon Buri | 0.333461 |
| 200905 | Samae San | Chon Buri | 0.328092 |
| 201001 | Bo Thong | Chon Buri | 0.255818 |
| 201004 | That Thong | Chon Buri | 0.274956 |
| 201102 | Tha Bunmi | Chon Buri | 0.340997 |
| 240101 | Na Mueang | Chachoengsao | 0.331008 |
| 240102 | Tha Khai | Chachoengsao | 0.347972 |
| 240109 | Bang Khwan | Chachoengsao | 0.3529 |
| 240110 | Khlong Nakhon Nueang Khet | Chachoengsao | 0.280198 |
| 240111 | Wang Takhian | Chachoengsao | 0.369384 |
| 240115 | Nam Daeng | Chachoengsao | 0.305473 |
| 240116 | Khlong Preng | Chachoengsao | 0.291821 |
| 240117 | Khlong Udom Chonlachon | Chachoengsao | 0.21163 |
| 240118 | Khlong Luang Phaeng | Chachoengsao | 0.273828 |
| 240119 | Bang Toei | Chachoengsao | 0.389167 |
| 240208 | Bang Krachet | Chachoengsao | 0.352397 |
| 240301 | Bang Nam Priao | Chachoengsao | 0.259671 |
| 240302 | Bang Khanak | Chachoengsao | 0.344052 |
| 240303 | Singto Thong | Chachoengsao | 0.309621 |
| 240304 | Mon Thong | Chachoengsao | 0.276867 |
| 240305 | Bueng Nam Rak | Chachoengsao | 0.368281 |
| 240306 | Don Ko Ka | Chachoengsao | 0.28695 |
| 240308 | Don Chimphli | Chachoengsao | 0.331629 |
| 240309 | Sala Daeng | Chachoengsao | 0.355591 |
| 240310 | Phrong Akat | Chachoengsao | 0.258872 |
| 240401 | Bang Pakong | Chachoengsao | 0.384769 |
| 240403 | Bang Wua | Chachoengsao | 0.392526 |
| 240404 | Bang Samak | Chachoengsao | 0.361001 |
| 240406 | Bang Kluea | Chachoengsao | 0.349481 |
| 240407 | Song Khlong | Chachoengsao | 0.275582 |
| 240411 | Hom Sin | Chachoengsao | 0.32351 |
| 240513 | Laem Pradu | Chachoengsao | 0.329468 |
| 240517 | Sip Et Sok | Chachoengsao | 0.329965 |
| 240601 | Ko Khanun | Chachoengsao | 0.250191 |
| 240602 | Ban Song | Chachoengsao | 0.369611 |
| 240603 | Phanom Sarakham | Chachoengsao | 0.318213 |
| 240604 | Mueang Kao | Chachoengsao | 0.34515 |
| 240605 | Nong Yao | Chachoengsao | 0.212149 |
| 240606 | Tha Than | Chachoengsao | 0.272218 |
| 240607 | Nong Nae | Chachoengsao | 0.328508 |
| 240703 | Dong Noi | Chachoengsao | 0.273651 |
| 240901 | Plaeng Yao | Chachoengsao | 0.312163 |
| 240902 | Wang Yen | Chachoengsao | 0.389653 |
| 240903 | Hua Samrong | Chachoengsao | 0.315749 |
| 241104 | Bang Rong | Chachoengsao | 0.366694 |
| 250101 | Na Mueang | Prachin Buri | 0.367093 |
| 250109 | Khok Mai Lai | Prachin Buri | 0.362936 |
| 250110 | Mai Khet | Prachin Buri | 0.38725 |
| 250111 | Dong Khi Lek | Prachin Buri | 0.358496 |
| 250112 | Noen Hom | Prachin Buri | 0.273237 |
| 250113 | Non Hom | Prachin Buri | 0.381291 |
| 250203 | Wang Dan | Prachin Buri | 0.255133 |
| 250207 | Hat Nang Kaeo | Prachin Buri | 0.221949 |
| 250209 | Ban Na | Prachin Buri | 0.205289 |
| 250606 | Bang Phluang | Prachin Buri | 0.385598 |
| 250607 | Bang Pla Ra | Prachin Buri | 0.313095 |
| 250609 | Krathum Phaeo | Prachin Buri | 0.2241 |
| 250701 | Prachantakham | Prachin Buri | 0.345202 |
| 250702 | Ko Loi | Prachin Buri | 0.21476 |
| 250704 | Nong Saeng | Prachin Buri | 0.21234 |
| 250708 | Nong Kaeo | Prachin Buri | 0.246866 |
| 250802 | Samphan | Prachin Buri | 0.313235 |
| 250803 | Ban Tham | Prachin Buri | 0.220286 |
| 250805 | Bang Kung | Prachin Buri | 0.288218 |
| 250806 | Dong Krathong Yam | Prachin Buri | 0.293642 |
| 250809 | Hat Yang | Prachin Buri | 0.360174 |
| 250903 | Khu Lamphan | Prachin Buri | 0.229281 |
| 250904 | Phai Chalueat | Prachin Buri | 0.268425 |
| 260101 | Nakhon Nayok | Nakhon Nayok | 0.324592 |
| 260102 | Tha Chang | Nakhon Nayok | 0.396905 |
| 260103 | Ban Yai | Nakhon Nayok | 0.308407 |
| 260104 | Wang Krachom | Nakhon Nayok | 0.232075 |
| 260106 | Don Yo | Nakhon Nayok | 0.296079 |
| 260107 | Si Chula | Nakhon Nayok | 0.325056 |
| 260108 | Dong Lakhon | Nakhon Nayok | 0.272803 |
| 260109 | Si Nawa | Nakhon Nayok | 0.31044 |
| 260112 | Khao Phra | Nakhon Nayok | 0.295112 |
| 260113 | Phrommani | Nakhon Nayok | 0.348838 |
| 260201 | Ko Wai | Nakhon Nayok | 0.302771 |
| 260202 | Ko Pho | Nakhon Nayok | 0.250031 |
| 260203 | Pak Phli | Nakhon Nayok | 0.360585 |
| 260204 | Khok Kruat | Nakhon Nayok | 0.333108 |
| 260205 | Tha Ruea | Nakhon Nayok | 0.316573 |
| 260206 | Nong Saeng | Nakhon Nayok | 0.299566 |
| 260401 | Phra Achan | Nakhon Nayok | 0.279884 |
| 260402 | Bueng San | Nakhon Nayok | 0.265053 |
| 260403 | Sisa Krabue | Nakhon Nayok | 0.215982 |
| 260404 | Pho Thaen | Nakhon Nayok | 0.362825 |
| 260406 | Sai Mun | Nakhon Nayok | 0.322108 |
| 260407 | Bang Pla Kot | Nakhon Nayok | 0.344402 |
| 260408 | Bang Luk Suea | Nakhon Nayok | 0.280029 |
| 260409 | Ongkharak | Nakhon Nayok | 0.2645 |
| 260410 | Chumphon | Nakhon Nayok | 0.361268 |
| 260411 | Khlong Yai | Nakhon Nayok | 0.280809 |
| 610105 | Hat Thanong | Uthai Thani | 0.205536 |
| 610106 | Ko Thepho | Uthai Thani | 0.20513 |
| 700104 | Nong Klang Na | Ratchaburi | 0.273479 |
| 700105 | Huai Phai | Ratchaburi | 0.349427 |
| 700106 | Khung Nam Won | Ratchaburi | 0.394247 |
| 700107 | Khung Krathin | Ratchaburi | 0.37312 |
| 700112 | Namphu | Ratchaburi | 0.390331 |
| 700117 | Lum Din | Ratchaburi | 0.231883 |
| 700118 | Bang Pa | Ratchaburi | 0.340458 |
| 700119 | Phong Sawai | Ratchaburi | 0.331107 |
| 700120 | Khu Bua | Ratchaburi | 0.251064 |
| 700121 | Tha Rap | Ratchaburi | 0.326216 |
| 700122 | Ban Rai | Ratchaburi | 0.246413 |
| 700201 | Chom Bueng | Ratchaburi | 0.370419 |
| 700202 | Pak Chong | Ratchaburi | 0.20824 |
| 700203 | Boek Phrai | Ratchaburi | 0.35494 |
| 700405 | Don Kruai | Ratchaburi | 0.366113 |
| 700406 | Don Khlang | Ratchaburi | 0.324923 |
| 700407 | Bua Ngam | Ratchaburi | 0.354759 |
| 700409 | Phaeng Phuai | Ratchaburi | 0.329697 |
| 700506 | Nong O | Ratchaburi | 0.206919 |
| 700507 | Don Krabueang | Ratchaburi | 0.232768 |
| 700508 | Suan Kluai | Ratchaburi | 0.249051 |
| 700509 | Nakhon Chum | Ratchaburi | 0.245884 |
| 700513 | Khao Khlung | Ratchaburi | 0.333608 |
| 700601 | Bang Phae | Ratchaburi | 0.223607 |
| 700603 | Hua Pho | Ratchaburi | 0.29344 |
| 700605 | Don Yai | Ratchaburi | 0.389073 |
| 700606 | Don Kha | Ratchaburi | 0.211572 |
| 700607 | Pho Hak | Ratchaburi | 0.249766 |
| 700701 | Photharam | Ratchaburi | 0.214438 |
| 700706 | Ban Khong | Ratchaburi | 0.332697 |
| 700707 | Ban Sing | Ratchaburi | 0.3771 |
| 700708 | Don Sai | Ratchaburi | 0.391023 |
| 700709 | Chet Samian | Ratchaburi | 0.255372 |
| 700710 | Khlong Khoi | Ratchaburi | 0.248367 |
| 700713 | Tha Chumphon | Ratchaburi | 0.36715 |
| 700714 | Bang Tanot | Ratchaburi | 0.24325 |
| 700715 | Tao Pun | Ratchaburi | 0.214372 |
| 700718 | Khao Cha-Ngum | Ratchaburi | 0.264917 |
| 700719 | Nong Kwang | Ratchaburi | 0.237023 |
| 700801 | Thung Luang | Ratchaburi | 0.38182 |
| 700802 | Wang Manao | Ratchaburi | 0.282677 |
| 700803 | Don Sai | Ratchaburi | 0.346115 |
| 700804 | Nong Krathum | Ratchaburi | 0.232248 |
| 700812 | Huai Yang Thon | Ratchaburi | 0.380731 |
| 700901 | Ko San Phra | Ratchaburi | 0.290969 |
| 700902 | Chom Prathat | Ratchaburi | 0.2226 |
| 700903 | Wat Phleng | Ratchaburi | 0.302927 |
| 710501 | Phong Tuek | Kanchanaburi | 0.201958 |
| 710509 | Don Khamin | Kanchanaburi | 0.201556 |
| 710611 | Tha Takhro | Kanchanaburi | 0.226077 |
| 711004 | Nong Pling | Kanchanaburi | 0.319469 |
| 711007 | Nong Fai | Kanchanaburi | 0.240594 |
| 720101 | Tha Phi Liang | Suphan Buri | 0.382971 |
| 720106 | Khok Kho Thao | Suphan Buri | 0.323041 |
| 720107 | Don Tan | Suphan Buri | 0.393125 |
| 720110 | Don Kamyan | Suphan Buri | 0.323123 |
| 720111 | Don Pho Thong | Suphan Buri | 0.34616 |
| 720114 | Taling Chan | Suphan Buri | 0.37929 |
| 720116 | Sala Khao | Suphan Buri | 0.288724 |
| 720117 | Suan Taeng | Suphan Buri | 0.319235 |
| 720201 | Khao Phra | Suphan Buri | 0.226981 |
| 720202 | Doem Bang | Suphan Buri | 0.24342 |
| 720203 | Nang Buat | Suphan Buri | 0.230555 |
| 720205 | Pak Nam | Suphan Buri | 0.29417 |
| 720208 | Hua Khao | Suphan Buri | 0.249257 |
| 720211 | Wang Si Rat | Suphan Buri | 0.254068 |
| 720214 | Nong Krathum | Suphan Buri | 0.226789 |
| 720301 | Nong Makha Mong | Suphan Buri | 0.354101 |
| 720302 | Dan Chang | Suphan Buri | 0.299523 |
| 720303 | Huai Khamin | Suphan Buri | 0.233447 |
| 720305 | Wang Khan | Suphan Buri | 0.317736 |
| 720306 | Nikhom Krasiao | Suphan Buri | 0.298111 |
| 720401 | Khok Khram | Suphan Buri | 0.362783 |
| 720407 | Phai Kong Din | Suphan Buri | 0.381113 |
| 720408 | Ongkharak | Suphan Buri | 0.316373 |
| 720409 | Chorakhe Yai | Suphan Buri | 0.238828 |
| 720411 | Makham Lom | Suphan Buri | 0.357565 |
| 720412 | Wang Nam Yen | Suphan Buri | 0.293657 |
| 720413 | Wat Bot | Suphan Buri | 0.344471 |
| 720414 | Wat Dao | Suphan Buri | 0.396916 |
| 720505 | Don Pru | Suphan Buri | 0.241618 |
| 720506 | Plai Na | Suphan Buri | 0.328752 |
| 720601 | Don Chedi | Suphan Buri | 0.383453 |
| 720602 | Nong Sarai | Suphan Buri | 0.341835 |
| 720603 | Rai Rot | Suphan Buri | 0.320376 |
| 720701 | Song Phi Nong | Suphan Buri | 0.300688 |
| 720702 | Bang Len | Suphan Buri | 0.312974 |
| 720703 | Bang Ta Then | Suphan Buri | 0.38943 |
| 720706 | Hua Pho | Suphan Buri | 0.361181 |
| 720707 | Bang Phlap | Suphan Buri | 0.351158 |
| 720708 | Noen Phraprang | Suphan Buri | 0.261617 |
| 720709 | Ban Chang | Suphan Buri | 0.39551 |
| 720710 | Ton Tan | Suphan Buri | 0.350796 |
| 720711 | Si Samran | Suphan Buri | 0.232563 |
| 720801 | Yan Yao | Suphan Buri | 0.366131 |
| 720803 | Sam Chuk | Suphan Buri | 0.28255 |
| 720805 | Ban Sa | Suphan Buri | 0.212299 |
| 720807 | Krasiao | Suphan Buri | 0.208177 |
| 720904 | Ban Don | Suphan Buri | 0.242037 |
| 720905 | Yung Thalai | Suphan Buri | 0.264874 |
| 720906 | Don Makluea | Suphan Buri | 0.249537 |
| 720908 | Don Kha | Suphan Buri | 0.248754 |
| 720911 | Chedi | Suphan Buri | 0.275056 |
| 720912 | Sa Phang Lan | Suphan Buri | 0.237443 |
| 720913 | Krachan | Suphan Buri | 0.268621 |
| 721004 | Chaeng Ngam | Suphan Buri | 0.32193 |
| 721005 | Nong Kham | Suphan Buri | 0.358518 |
| 721006 | Thap Luang | Suphan Buri | 0.219663 |
| 730102 | Bang Khaem | Nakhon Pathom | 0.326613 |
| 730103 | Phra Prathon | Nakhon Pathom | 0.292143 |
| 730108 | Don Yai Hom | Nakhon Pathom | 0.204499 |
| 730110 | Bo Phlap | Nakhon Pathom | 0.269414 |
| 730112 | Wang Taku | Nakhon Pathom | 0.343619 |
| 730113 | Nong Pak Long | Nakhon Pathom | 0.35873 |
| 730114 | Sam Khwai Phueak | Nakhon Pathom | 0.315476 |
| 730115 | Thung Noi | Nakhon Pathom | 0.335445 |
| 730116 | Nong Din Daeng | Nakhon Pathom | 0.322011 |
| 730118 | Phrong Maduea | Nakhon Pathom | 0.355062 |
| 730120 | Sa Kathiam | Nakhon Pathom | 0.21345 |
| 730123 | Thap Luang | Nakhon Pathom | 0.376896 |
| 730124 | Nong Ngu Lueam | Nakhon Pathom | 0.277623 |
| 730201 | Thung Kraphang Hom | Nakhon Pathom | 0.240986 |
| 730203 | Thung Luk Nok | Nakhon Pathom | 0.239479 |
| 730204 | Huai Khwang | Nakhon Pathom | 0.281215 |
| 730206 | Sa Si Mum | Nakhon Pathom | 0.234348 |
| 730208 | Don Khoi | Nakhon Pathom | 0.291898 |
| 730209 | Sa Phatthana | Nakhon Pathom | 0.220254 |
| 730211 | Huai Muang | Nakhon Pathom | 0.208637 |
| 730215 | Wang Nam Khiao | Nakhon Pathom | 0.207247 |
| 730306 | Tha Krachap | Nakhon Pathom | 0.214221 |
| 730307 | Khun Kaeo | Nakhon Pathom | 0.224714 |
| 730313 | Laem Bua | Nakhon Pathom | 0.235626 |
| 730314 | Si Mahapho | Nakhon Pathom | 0.255906 |
| 730315 | Sampathuan | Nakhon Pathom | 0.244481 |
| 730316 | Wat Samrong | Nakhon Pathom | 0.242541 |
| 730318 | Huai Phlu | Nakhon Pathom | 0.229768 |
| 730319 | Wat Lamut | Nakhon Pathom | 0.264801 |
| 730320 | Bang Phra | Nakhon Pathom | 0.306752 |
| 730321 | Bang Kaeo Fa | Nakhon Pathom | 0.330242 |
| 730322 | Lan Tak Fa | Nakhon Pathom | 0.236426 |
| 730323 | Ngio Rai | Nakhon Pathom | 0.279163 |
| 730324 | Thaiyawat | Nakhon Pathom | 0.276684 |
| 730401 | Sam Ngam | Nakhon Pathom | 0.338725 |
| 730402 | Huai Phra | Nakhon Pathom | 0.253327 |
| 730403 | Lam Hoei | Nakhon Pathom | 0.260151 |
| 730404 | Don Phutsa | Nakhon Pathom | 0.314371 |
| 730405 | Ban Luang | Nakhon Pathom | 0.268858 |
| 730406 | Don Ruak | Nakhon Pathom | 0.274094 |
| 730407 | Huai Duan | Nakhon Pathom | 0.232186 |
| 730408 | Lam Luk Bua | Nakhon Pathom | 0.35954 |
| 730503 | Bang Luang | Nakhon Pathom | 0.382201 |
| 730505 | Bang Rakam | Nakhon Pathom | 0.334521 |
| 730510 | Nin Phet | Nakhon Pathom | 0.396183 |
| 730511 | Bua Pak Tha | Nakhon Pathom | 0.366471 |
| 730513 | Nara Phirom | Nakhon Pathom | 0.353169 |
| 730515 | Phai Hu Chang | Nakhon Pathom | 0.378329 |
| 730601 | Tha Kham | Nakhon Pathom | 0.398474 |
| 730602 | Song Khanong | Nakhon Pathom | 0.200947 |
| 730603 | Hom Kret | Nakhon Pathom | 0.228873 |
| 730605 | Bang Toei | Nakhon Pathom | 0.20297 |
| 730607 | Bang Chang | Nakhon Pathom | 0.243083 |
| 730608 | Rai Khing | Nakhon Pathom | 0.222457 |
| 730610 | Krathum Lom | Nakhon Pathom | 0.20024 |
| 730612 | Talat Chinda | Nakhon Pathom | 0.252504 |
| 730614 | Yai Cha | Nakhon Pathom | 0.263492 |
| 730702 | Khlong Yong | Nakhon Pathom | 0.212882 |
| 740103 | Krok Krak | Samut Sakhon | 0.242917 |
| 740104 | Ban Bo | Samut Sakhon | 0.353012 |
| 740108 | Tha Chin | Samut Sakhon | 0.251149 |
| 740110 | Tha Sai | Samut Sakhon | 0.231156 |
| 740112 | Bang Nam Chuet | Samut Sakhon | 0.202015 |
| 740115 | Ban Ko | Samut Sakhon | 0.342114 |
| 740116 | Bang Krachao | Samut Sakhon | 0.370195 |
| 740118 | Chai Mongkhon | Samut Sakhon | 0.38164 |
| 740201 | Talat Krathum Baen | Samut Sakhon | 0.271128 |
| 740203 | Tha Mai | Samut Sakhon | 0.253465 |
| 740205 | Bang Yang | Samut Sakhon | 0.328447 |
| 740207 | Nong Nok Khai | Samut Sakhon | 0.258031 |
| 740210 | Tha Sao | Samut Sakhon | 0.25167 |
| 740301 | Ban Phaeo | Samut Sakhon | 0.37925 |
| 740307 | Lak Song | Samut Sakhon | 0.391865 |
| 740308 | Chet Rio | Samut Sakhon | 0.30969 |
| 740309 | Khlong Tan | Samut Sakhon | 0.290525 |
| 740310 | Amphaeng | Samut Sakhon | 0.340986 |
| 740311 | Suan Som | Samut Sakhon | 0.371557 |
| 740312 | Kaset Phatthana | Samut Sakhon | 0.23598 |
| 750101 | Mae Klong | Samut Songkhram | 0.390972 |
| 750102 | Bang Khan Taek | Samut Songkhram | 0.391259 |
| 750107 | Laem Yai | Samut Songkhram | 0.33064 |
| 750109 | Khlong Khon | Samut Songkhram | 0.220723 |
| 750111 | Bang Chakreng | Samut Songkhram | 0.343408 |
| 750304 | Wat Pradu | Samut Songkhram | 0.243859 |
| 750308 | Plai Phongphang | Samut Songkhram | 0.297857 |
| 750309 | Bang Khae | Samut Songkhram | 0.350272 |
| 750312 | Bang Nang Li | Samut Songkhram | 0.348607 |
| 760106 | Ban Kum | Phetchaburi | 0.229724 |
| 760107 | Nong Sano | Phetchaburi | 0.258459 |
| 760210 | Nong Chumphon Nuea | Phetchaburi | 0.221597 |
| 760301 | Nong Ya Plong | Phetchaburi | 0.203266 |
| 760304 | Tha Takhro | Phetchaburi | 0.204756 |
| 760502 | Tha Khoi | Phetchaburi | 0.276129 |
| 760503 | Yang Yong | Phetchaburi | 0.237135 |
| 760601 | Ban Lat | Phetchaburi | 0.288327 |
| 760604 | Tamru | Phetchaburi | 0.283257 |
| 760605 | Samo Phlue | Phetchaburi | 0.274318 |
| 760607 | Tha Sen | Phetchaburi | 0.31124 |
| 760616 | Tha Chang | Phetchaburi | 0.203748 |
| 760617 | Tham Rong | Phetchaburi | 0.277035 |
| 760706 | Bang Tabun | Phetchaburi | 0.220837 |
| 760707 | Bang Tabun Ok | Phetchaburi | 0.262757 |
| 760708 | Bang Khrok | Phetchaburi | 0.224885 |

**Table S4** The list of very low risk subdistricts for NiV occurrence at subdistrict level

| **Geocode** | **Name of the subdistrict** | **Province** | **Probability of NiV occurrence** |
| --- | --- | --- | --- |
| 100105 | San Chaopho Suea | Bangkok | 0.195434 |
| 100107 | Bowon Niwet | Bangkok | 0.198588 |
| 100108 | Talat Yot | Bangkok | 0.182127 |
| 100110 | Ban Phan Thom | Bangkok | 0.192792 |
| 100111 | Bang Khun Phrom | Bangkok | 0.19517 |
| 100201 | Dusit | Bangkok | 0.118129 |
| 100203 | Suan Chitlada | Bangkok | 0.113487 |
| 100206 | Thanon Nakhon Chaisi | Bangkok | 0.124606 |
| 100502 | Anusawari | Bangkok | 0.089426 |
| 100601 | Khlong Chan | Bangkok | 0.137174 |
| 100608 | Hua Mak | Bangkok | 0.190594 |
| 100702 | Wang Mai | Bangkok | 0.15671 |
| 100703 | Pathum Wan | Bangkok | 0.156256 |
| 100704 | Lumphini | Bangkok | 0.189643 |
| 101101 | Lat Krabang | Bangkok | 0.199932 |
| 101401 | Sam Sen Nai | Bangkok | 0.100372 |
| 101602 | Wat Tha Phra | Bangkok | 0.197809 |
| 101701 | Huai Khwang | Bangkok | 0.140914 |
| 101704 | Sam Sen Nok | Bangkok | 0.149093 |
| 101801 | Somdet Chaophraya | Bangkok | 0.185298 |
| 101902 | Taling Chan | Bangkok | 0.148377 |
| 101903 | Chimphli | Bangkok | 0.130228 |
| 102004 | Sirirat | Bangkok | 0.199797 |
| 102005 | Ban Chang Lo | Bangkok | 0.176879 |
| 102006 | Bang Khun Non | Bangkok | 0.173193 |
| 102007 | Bang Khun Si | Bangkok | 0.189155 |
| 102009 | Arun Ammarin | Bangkok | 0.14367 |
| 102202 | Bang Duan | Bangkok | 0.185781 |
| 102302 | Nong Khaem | Bangkok | 0.180204 |
| 102303 | Nong Khang Phlu | Bangkok | 0.171228 |
| 102501 | Bang Phlat | Bangkok | 0.137442 |
| 102502 | Bang O | Bangkok | 0.150374 |
| 102503 | Bang Bamru | Bangkok | 0.077558 |
| 102504 | Bang Yi Khan | Bangkok | 0.154127 |
| 102601 | Din Daeng | Bangkok | 0.136893 |
| 102701 | Khlong Kum | Bangkok | 0.170336 |
| 102901 | Bang Sue | Bangkok | 0.089141 |
| 103001 | Lat Yao | Bangkok | 0.043108 |
| 103002 | Sena Nikhom | Bangkok | 0.050744 |
| 103003 | Chantharakasem | Bangkok | 0.051642 |
| 103004 | Chomphon | Bangkok | 0.055282 |
| 103005 | Chatuchak | Bangkok | 0.045644 |
| 103602 | Si Kan | Bangkok | 0.087777 |
| 103701 | Thung Phaya Thai | Bangkok | 0.111708 |
| 103702 | Thanon Phaya Thai | Bangkok | 0.153623 |
| 103703 | Thanon Phet Buri | Bangkok | 0.17861 |
| 103704 | Makkasan | Bangkok | 0.184516 |
| 103801 | Lat Phrao | Bangkok | 0.077886 |
| 103802 | Chorakhe Bua | Bangkok | 0.099753 |
| 104001 | Bang Khae | Bangkok | 0.15159 |
| 104002 | Bang Khae Nuea | Bangkok | 0.143561 |
| 104003 | Bang Phai | Bangkok | 0.163218 |
| 104004 | Lak Song | Bangkok | 0.15933 |
| 104101 | Thung Song Hong | Bangkok | 0.052705 |
| 104102 | Talat Bang Khen | Bangkok | 0.056385 |
| 104203 | Khlong Thanon | Bangkok | 0.183167 |
| 104501 | Wang Thong Lang | Bangkok | 0.132551 |
| 104801 | Thawi Watthana | Bangkok | 0.16099 |
| 104802 | Sala Thammasop | Bangkok | 0.096401 |
| 105001 | Bang Bon | Bangkok | 0.157834 |
| 110309 | Nong Prue | Samut Prakan | 0.163367 |
| 120102 | Talat Khwan | Nonthaburi | 0.085716 |
| 120103 | Bang Khen | Nonthaburi | 0.074629 |
| 120104 | Bang Kraso | Nonthaburi | 0.073786 |
| 120105 | Tha Sai | Nonthaburi | 0.041495 |
| 120109 | Sai Ma | Nonthaburi | 0.17402 |
| 120202 | Bang Kruai | Nonthaburi | 0.148363 |
| 120302 | Bang Mae Nang | Nonthaburi | 0.182368 |
| 120304 | Sao Thong Hin | Nonthaburi | 0.123658 |
| 120306 | Ban Mai | Nonthaburi | 0.160403 |
| 120401 | Sano Loi | Nonthaburi | 0.17101 |
| 120407 | Phimonrat | Nonthaburi | 0.189975 |
| 120408 | Bang Rak Phatthana | Nonthaburi | 0.132715 |
| 120601 | Pak Kret | Nonthaburi | 0.086667 |
| 120602 | Bang Talat | Nonthaburi | 0.035446 |
| 120603 | Ban Mai | Nonthaburi | 0.097138 |
| 120604 | Bang Phut | Nonthaburi | 0.135155 |
| 120612 | Khlong Kluea | Nonthaburi | 0.036953 |
| 130102 | Ban Mai | Pathum Thani | 0.169952 |
| 130114 | Lak Hok | Pathum Thani | 0.198389 |
| 130601 | Khu Khot | Pathum Thani | 0.154577 |
| 140807 | Don Lan | Phra Nakhon Si Ayutthaya | 0.189924 |
| 140810 | Lam Takhian | Phra Nakhon Si Ayutthaya | 0.140777 |
| 140812 | Chakkarat | Phra Nakhon Si Ayutthaya | 0.138948 |
| 140813 | Nong Nam Yai | Phra Nakhon Si Ayutthaya | 0.162281 |
| 140814 | Lat Chit | Phra Nakhon Si Ayutthaya | 0.142986 |
| 141209 | Rang Chorakhe | Phra Nakhon Si Ayutthaya | 0.157087 |
| 141212 | Chai Na | Phra Nakhon Si Ayutthaya | 0.196819 |
| 141214 | Lat Nga | Phra Nakhon Si Ayutthaya | 0.107811 |
| 141215 | Don Thong | Phra Nakhon Si Ayutthaya | 0.195522 |
| 141217 | Chao Sadet | Phra Nakhon Si Ayutthaya | 0.190515 |
| 141304 | Plai Klat | Phra Nakhon Si Ayutthaya | 0.18767 |
| 160101 | Thale Chup Son | Lop Buri | 0.187556 |
| 160102 | Tha Hin | Lop Buri | 0.143012 |
| 160103 | Kok Ko | Lop Buri | 0.134681 |
| 160105 | Khao Phra Ngam | Lop Buri | 0.136302 |
| 160106 | Khao Sam Yot | Lop Buri | 0.0804 |
| 160108 | Khok Lamphan | Lop Buri | 0.195097 |
| 160114 | Tha Khae | Lop Buri | 0.176839 |
| 160115 | Tha Sala | Lop Buri | 0.124827 |
| 160116 | Nikhom Sang Ton-Eng | Lop Buri | 0.147402 |
| 160120 | Pa Tan | Lop Buri | 0.161088 |
| 160125 | Thanon Yai | Lop Buri | 0.175271 |
| 160201 | Phatthana Nikhom | Lop Buri | 0.193129 |
| 160202 | Chong Sarika | Lop Buri | 0.139461 |
| 160204 | Di Lang | Lop Buri | 0.122783 |
| 160206 | Chon Noi | Lop Buri | 0.131014 |
| 160208 | Huai Khun Ram | Lop Buri | 0.098478 |
| 160209 | Nam Sut | Lop Buri | 0.089828 |
| 160301 | Khok Samrong | Lop Buri | 0.059418 |
| 160302 | Ko Kaeo | Lop Buri | 0.086093 |
| 160303 | Thalung Lek | Lop Buri | 0.098627 |
| 160304 | Lum Khao | Lop Buri | 0.094099 |
| 160305 | Huai Pong | Lop Buri | 0.11753 |
| 160306 | Khlong Ket | Lop Buri | 0.09001 |
| 160307 | Sakae Rap | Lop Buri | 0.094382 |
| 160309 | Wang Phloeng | Lop Buri | 0.119114 |
| 160310 | Dong Marum | Lop Buri | 0.116198 |
| 160318 | Wang Khon Khwang | Lop Buri | 0.085763 |
| 160320 | Wang Chan | Lop Buri | 0.095958 |
| 160322 | Nong Khaem | Lop Buri | 0.108582 |
| 160401 | Lam Narai | Lop Buri | 0.133388 |
| 160402 | Chai Narai | Lop Buri | 0.141504 |
| 160403 | Sila Thip | Lop Buri | 0.198674 |
| 160404 | Huai Hin | Lop Buri | 0.137589 |
| 160405 | Muang Khom | Lop Buri | 0.119093 |
| 160406 | Bua Chum | Lop Buri | 0.106257 |
| 160408 | Makok Wan | Lop Buri | 0.133139 |
| 160409 | Sap Takhian | Lop Buri | 0.105605 |
| 160410 | Na Som | Lop Buri | 0.124698 |
| 160411 | Nong Yai To | Lop Buri | 0.121066 |
| 160412 | Ko Rang | Lop Buri | 0.130254 |
| 160417 | Nikhom Lam Narai | Lop Buri | 0.121752 |
| 160418 | Chai Badan | Lop Buri | 0.160477 |
| 160419 | Ban Mai Samakkhi | Lop Buri | 0.151468 |
| 160422 | Khao Laem | Lop Buri | 0.129965 |
| 160601 | Phai Yai | Lop Buri | 0.096566 |
| 160602 | Ban Sai | Lop Buri | 0.087317 |
| 160603 | Ban Kluai | Lop Buri | 0.143948 |
| 160604 | Dong Phlap | Lop Buri | 0.100127 |
| 160606 | Phu Kha | Lop Buri | 0.089293 |
| 160607 | Hin Pak | Lop Buri | 0.08435 |
| 160608 | Bang Phueng | Lop Buri | 0.179519 |
| 160609 | Nong Sai Khao | Lop Buri | 0.135689 |
| 160610 | Bang Kaphi | Lop Buri | 0.095737 |
| 160611 | Nong Tao | Lop Buri | 0.119152 |
| 160612 | Phon Thong | Lop Buri | 0.086275 |
| 160614 | Don Dueng | Lop Buri | 0.131195 |
| 160616 | Nong Krabian | Lop Buri | 0.098699 |
| 160617 | Sai Huai Kaeo | Lop Buri | 0.10851 |
| 160618 | Mahason | Lop Buri | 0.148489 |
| 160619 | Ban Mi | Lop Buri | 0.063071 |
| 160620 | Chiang Nga | Lop Buri | 0.096747 |
| 160621 | Nong Mueang | Lop Buri | 0.088227 |
| 160622 | Sanam Chaeng | Lop Buri | 0.106037 |
| 160701 | Tha Luang | Lop Buri | 0.143276 |
| 160702 | Kaeng Phak Kut | Lop Buri | 0.10745 |
| 160703 | Sap Champa | Lop Buri | 0.092627 |
| 160704 | Nong Phak Waen | Lop Buri | 0.100026 |
| 160705 | Thale Wang Wat | Lop Buri | 0.088641 |
| 160706 | Hua Lam | Lop Buri | 0.112389 |
| 160801 | Sa Bot | Lop Buri | 0.105826 |
| 160802 | Mahapho | Lop Buri | 0.133435 |
| 160803 | Thung Tha Chang | Lop Buri | 0.097642 |
| 160804 | Huai Yai | Lop Buri | 0.096658 |
| 160805 | Niyom Chai | Lop Buri | 0.131567 |
| 160901 | Khok Charoen | Lop Buri | 0.090042 |
| 160902 | Yang Rak | Lop Buri | 0.131463 |
| 160903 | Nong Makha | Lop Buri | 0.102776 |
| 160904 | Wang Thong | Lop Buri | 0.113047 |
| 160905 | Khok Samae San | Lop Buri | 0.100773 |
| 161001 | Lam Sonthi | Lop Buri | 0.135611 |
| 161002 | Sap Sombun | Lop Buri | 0.09394 |
| 161003 | Nong Ri | Lop Buri | 0.126112 |
| 161004 | Kut Ta Phet | Lop Buri | 0.139743 |
| 161005 | Khao Ruak | Lop Buri | 0.121168 |
| 161006 | Khao Noi | Lop Buri | 0.118611 |
| 161101 | Nong Muang | Lop Buri | 0.130882 |
| 161102 | Bo Thong | Lop Buri | 0.130314 |
| 161103 | Dong Din Daeng | Lop Buri | 0.095371 |
| 161104 | Chon Sombun | Lop Buri | 0.102459 |
| 161105 | Yang Thon | Lop Buri | 0.128515 |
| 161106 | Chon Saradet | Lop Buri | 0.099328 |
| 170105 | Hua Phai | Sing Buri | 0.187955 |
| 170205 | Mae La | Sing Buri | 0.157237 |
| 170207 | Phak Than | Sing Buri | 0.133764 |
| 170208 | Sa Chaeng | Sing Buri | 0.126706 |
| 170301 | Pho Thale | Sing Buri | 0.167874 |
| 170306 | Nong Krathum | Sing Buri | 0.14763 |
| 170601 | In Buri | Sing Buri | 0.185037 |
| 170602 | Prasuk | Sing Buri | 0.167351 |
| 170604 | Ngio Rai | Sing Buri | 0.149961 |
| 170605 | Chi Nam Rai | Sing Buri | 0.193678 |
| 170608 | Thong En | Sing Buri | 0.113324 |
| 170609 | Huai Chan | Sing Buri | 0.124022 |
| 170610 | Pho Chai | Sing Buri | 0.10171 |
| 180108 | Suea Hok | Chai Nat | 0.112533 |
| 180202 | Wat Khok | Chai Nat | 0.144461 |
| 180203 | Sila Dan | Chai Nat | 0.181385 |
| 180204 | Tha Chanuan | Chai Nat | 0.169201 |
| 180205 | Hang Nam Sakhon | Chai Nat | 0.143705 |
| 180206 | Rai Phatthana | Chai Nat | 0.097117 |
| 180207 | U Taphao | Chai Nat | 0.140011 |
| 180307 | Bo Rae | Chai Nat | 0.164069 |
| 180402 | Taluk | Chai Nat | 0.198657 |
| 180403 | Khao Kaeo | Chai Nat | 0.101419 |
| 180407 | Hat Asa | Chai Nat | 0.151095 |
| 180508 | Huai Krot Phatthana | Chai Nat | 0.176721 |
| 180701 | Nong Mamong | Chai Nat | 0.129186 |
| 180702 | Wang Takhian | Chai Nat | 0.112216 |
| 180703 | Saphan Hin | Chai Nat | 0.152808 |
| 180704 | Kut Chok | Chai Nat | 0.198756 |
| 180801 | Noen Kham | Chai Nat | 0.163129 |
| 180802 | Kabok Tia | Chai Nat | 0.138031 |
| 180803 | Suk Duean Ha | Chai Nat | 0.164488 |
| 190201 | Kaeng Khoi | Saraburi | 0.136861 |
| 190205 | Tha Khlo | Saraburi | 0.178469 |
| 190206 | Hin Son | Saraburi | 0.146363 |
| 190208 | Ban Pa | Saraburi | 0.17946 |
| 190213 | Cham Phak Phaeo | Saraburi | 0.165052 |
| 190215 | Tha Maprang | Saraburi | 0.199531 |
| 190801 | Nong Don | Saraburi | 0.182713 |
| 190802 | Ban Klap | Saraburi | 0.118612 |
| 190803 | Don Thong | Saraburi | 0.171498 |
| 190901 | Phraphutthabat | Saraburi | 0.096976 |
| 190902 | Khun Khlon | Saraburi | 0.120983 |
| 190904 | Na Yao | Saraburi | 0.158733 |
| 190905 | Phu Kham Chan | Saraburi | 0.193685 |
| 190909 | Nong Kae | Saraburi | 0.136717 |
| 191101 | Muak Lek | Saraburi | 0.159956 |
| 191102 | Mittraphap | Saraburi | 0.143071 |
| 191104 | Nong Yang Suea | Saraburi | 0.153594 |
| 191105 | Lam Somphung | Saraburi | 0.132738 |
| 191107 | Lam Phaya Klang | Saraburi | 0.132643 |
| 191109 | Sap Sanun | Saraburi | 0.129426 |
| 191203 | Wang Muang | Saraburi | 0.121169 |
| 191306 | Na Phralan | Saraburi | 0.170728 |
| 200208 | Nong Phai Kaeo | Chon Buri | 0.169008 |
| 200301 | Nong Yai | Chon Buri | 0.158513 |
| 200302 | Khlong Phlu | Chon Buri | 0.130896 |
| 200303 | Nong Suea Chang | Chon Buri | 0.142712 |
| 200305 | Khao Sok | Chon Buri | 0.120398 |
| 200401 | Bang Lamung | Chon Buri | 0.161114 |
| 200402 | Nong Prue | Chon Buri | 0.077842 |
| 200403 | Nong Pla Lai | Chon Buri | 0.145477 |
| 200404 | Pong | Chon Buri | 0.149674 |
| 200405 | Khao Mai Kaeo | Chon Buri | 0.15459 |
| 200406 | Huai Yai | Chon Buri | 0.182235 |
| 200408 | Na Kluea | Chon Buri | 0.124166 |
| 200702 | Surasak | Chon Buri | 0.180619 |
| 200703 | Thung Sukhla | Chon Buri | 0.072182 |
| 200704 | Bueng | Chon Buri | 0.173875 |
| 200705 | Nong Kham | Chon Buri | 0.175693 |
| 200706 | Khao Khan Song | Chon Buri | 0.136748 |
| 200708 | Bo Win | Chon Buri | 0.121003 |
| 200901 | Sattahip | Chon Buri | 0.165621 |
| 200902 | Na Chom Thian | Chon Buri | 0.162098 |
| 200903 | Phlu Ta Luang | Chon Buri | 0.129174 |
| 200904 | Bang Sare | Chon Buri | 0.157774 |
| 201002 | Wat Suwan | Chon Buri | 0.185559 |
| 201005 | Kaset Suwan | Chon Buri | 0.193725 |
| 201006 | Phluang Thong | Chon Buri | 0.145867 |
| 201101 | Ko Chan | Chon Buri | 0.197683 |
| 210201 | Samnak Thon | Rayong | 0 |
| 210202 | Phla | Rayong | 0 |
| 210402 | Chum Saeng | Rayong | 0 |
| 210403 | Pa Yup Nai | Rayong | 0 |
| 210601 | Pluak Daeng | Rayong | 0 |
| 210602 | Ta Sit | Rayong | 0 |
| 210605 | Map Yang Phon | Rayong | 0 |
| 210606 | Nong Rai | Rayong | 0 |
| 210702 | Huai Thap Mon | Rayong | 0 |
| 210704 | Khao Noi | Rayong | 0 |
| 210803 | Phana Nikhom | Rayong | 0 |
| 210804 | Makham Khu | Rayong | 0 |
| 220802 | Khun Song | Chanthaburi | 0 |
| 220804 | Phawa | Chanthaburi | 0 |
| 240608 | Khao Hin Son | Chachoengsao | 0.140518 |
| 240801 | Khu Yai Mi | Chachoengsao | 0.156236 |
| 240802 | Tha Kradan | Chachoengsao | 0.120029 |
| 240803 | Thung Phraya | Chachoengsao | 0.112028 |
| 240805 | Lat Krathing | Chachoengsao | 0.128317 |
| 240904 | Nong Mai Kaen | Chachoengsao | 0.13617 |
| 241001 | Tha Takiap | Chachoengsao | 0.129629 |
| 241002 | Khlong Takrao | Chachoengsao | 0.136371 |
| 250201 | Kabin | Prachin Buri | 0.146303 |
| 250202 | Mueang Kao | Prachin Buri | 0.160653 |
| 250204 | Nonsi | Prachin Buri | 0.168425 |
| 250205 | Yan Ri | Prachin Buri | 0.103725 |
| 250206 | Wang Takhian | Prachin Buri | 0.119589 |
| 250208 | Lat Takhian | Prachin Buri | 0.119564 |
| 250210 | Bo Thong | Prachin Buri | 0.113653 |
| 250211 | Nong Ki | Prachin Buri | 0.134339 |
| 250212 | Na Khaem | Prachin Buri | 0.143298 |
| 250213 | Khao Mai Kaeo | Prachin Buri | 0.09675 |
| 250214 | Wang Tha Chang | Prachin Buri | 0.100362 |
| 250301 | Na Di | Prachin Buri | 0.174809 |
| 250302 | Sam Phan Ta | Prachin Buri | 0.140671 |
| 250303 | Saphan Hin | Prachin Buri | 0.149755 |
| 250304 | Thung Pho | Prachin Buri | 0.128063 |
| 250305 | Kaeng Dinso | Prachin Buri | 0.135544 |
| 250306 | Bu Phram | Prachin Buri | 0.131745 |
| 250703 | Ban Hoi | Prachin Buri | 0.190411 |
| 250705 | Dong Bang | Prachin Buri | 0.140585 |
| 250706 | Kham Tanot | Prachin Buri | 0.152854 |
| 250707 | Bu Fai | Prachin Buri | 0.156449 |
| 250709 | Pho Ngam | Prachin Buri | 0.171205 |
| 250801 | Si Mahapho | Prachin Buri | 0.129023 |
| 250804 | Tha Tum | Prachin Buri | 0.135039 |
| 250807 | Nong Phrong | Prachin Buri | 0.130949 |
| 250808 | Hua Wa | Prachin Buri | 0.129708 |
| 250810 | Krok Sombun | Prachin Buri | 0.129747 |
| 250901 | Khok Pip | Prachin Buri | 0.175115 |
| 250902 | Khok Thai | Prachin Buri | 0.140284 |
| 260110 | Sarika | Nakhon Nayok | 0.18866 |
| 260111 | Hin Tang | Nakhon Nayok | 0.156808 |
| 260207 | Na Hin Lat | Nakhon Nayok | 0.164573 |
| 270102 | Ban Kaeng | Sa Kaeo | 0 |
| 270103 | Sala Lamduan | Sa Kaeo | 0 |
| 270104 | Khok Pi Khong | Sa Kaeo | 0.126894 |
| 270108 | Sa Khwan | Sa Kaeo | 0 |
| 270406 | Thung Mahacharoen | Sa Kaeo | 0 |
| 270702 | Nong Wa | Sa Kaeo | 0 |
| 270703 | Phra Phloeng | Sa Kaeo | 0 |
| 270902 | Wang Mai | Sa Kaeo | 0 |
| 300205 | Chorakhe Hin | Nakhon Ratchasima | 0 |
| 300815 | Huai Bong | Nakhon Ratchasima | 0 |
| 302007 | Nong Nam Sai | Nakhon Ratchasima | 0 |
| 302011 | Don Mueang | Nakhon Ratchasima | 0 |
| 302101 | Pak Chong | Nakhon Ratchasima | 0 |
| 302102 | Klang Dong | Nakhon Ratchasima | 0 |
| 302103 | Chan Thuek | Nakhon Ratchasima | 0 |
| 302105 | Mu Si | Nakhon Ratchasima | 0 |
| 302108 | Pong Ta Long | Nakhon Ratchasima | 0 |
| 302112 | Phaya Yen | Nakhon Ratchasima | 0 |
| 302501 | Wang Nam Khiao | Nakhon Ratchasima | 0.138798 |
| 302502 | Wang Mi | Nakhon Ratchasima | 0 |
| 302505 | Thai Samakkhi | Nakhon Ratchasima | 0 |
| 302602 | Nong Waeng | Nakhon Ratchasima | 0 |
| 302603 | Bueng Prue | Nakhon Ratchasima | 0 |
| 360901 | Wa Tabaek | Chaiyaphum | 0 |
| 360902 | Huai Yai Chio | Chaiyaphum | 0 |
| 360904 | Ban Rai | Chaiyaphum | 0 |
| 600101 | Pak Nam Pho | Nakhon Sawan | 0.073978 |
| 600102 | Klang Daet | Nakhon Sawan | 0.125168 |
| 600103 | Kriangkrai | Nakhon Sawan | 0.131743 |
| 600104 | Khwae Yai | Nakhon Sawan | 0.173963 |
| 600105 | Takhian Luean | Nakhon Sawan | 0.121096 |
| 600106 | Nakhon Sawan Tok | Nakhon Sawan | 0.071846 |
| 600107 | Nakhon Sawan Ok | Nakhon Sawan | 0.092032 |
| 600108 | Bang Phra Luang | Nakhon Sawan | 0.137863 |
| 600109 | Bang Muang | Nakhon Sawan | 0.119936 |
| 600110 | Ban Makluea | Nakhon Sawan | 0.098787 |
| 600111 | Ban Kaeng | Nakhon Sawan | 0.109152 |
| 600112 | Phra Non | Nakhon Sawan | 0.116767 |
| 600113 | Wat Sai | Nakhon Sawan | 0.094249 |
| 600114 | Nong Krot | Nakhon Sawan | 0.071179 |
| 600115 | Nong Kradon | Nakhon Sawan | 0.070429 |
| 600116 | Nong Pling | Nakhon Sawan | 0.080237 |
| 600117 | Bueng Senat | Nakhon Sawan | 0.094285 |
| 600201 | Krok Phra | Nakhon Sawan | 0.11099 |
| 600202 | Yang Tan | Nakhon Sawan | 0.153604 |
| 600203 | Bang Mafo | Nakhon Sawan | 0.150798 |
| 600204 | Bang Pramung | Nakhon Sawan | 0.073903 |
| 600205 | Na Klang | Nakhon Sawan | 0.07061 |
| 600206 | Sala Daeng | Nakhon Sawan | 0.074392 |
| 600207 | Noen Kwao | Nakhon Sawan | 0.087133 |
| 600208 | Noen Sala | Nakhon Sawan | 0.099293 |
| 600209 | Hat Sung | Nakhon Sawan | 0.138469 |
| 600301 | Chum Saeng | Nakhon Sawan | 0.075294 |
| 600302 | Thap Krit | Nakhon Sawan | 0.101041 |
| 600303 | Phikun | Nakhon Sawan | 0.113349 |
| 600304 | Koei Chai | Nakhon Sawan | 0.138152 |
| 600305 | Tha Mai | Nakhon Sawan | 0.162045 |
| 600306 | Bang Khian | Nakhon Sawan | 0.125414 |
| 600307 | Nong Krachao | Nakhon Sawan | 0.072092 |
| 600308 | Phan Lan | Nakhon Sawan | 0.149329 |
| 600309 | Khok Mo | Nakhon Sawan | 0.150546 |
| 600310 | Phai Sing | Nakhon Sawan | 0.072929 |
| 600311 | Kha Mang | Nakhon Sawan | 0.13443 |
| 600312 | Thap Krit Tai | Nakhon Sawan | 0.165865 |
| 600401 | Nong Bua | Nakhon Sawan | 0.07788 |
| 600402 | Nong Klap | Nakhon Sawan | 0.066961 |
| 600403 | Than Thahan | Nakhon Sawan | 0.076314 |
| 600404 | Huai Ruam | Nakhon Sawan | 0.071964 |
| 600405 | Huai Thua Tai | Nakhon Sawan | 0.076783 |
| 600406 | Huai Thua Nuea | Nakhon Sawan | 0.070883 |
| 600407 | Huai Yai | Nakhon Sawan | 0.074394 |
| 600408 | Thung Thong | Nakhon Sawan | 0.070563 |
| 600409 | Wang Bo | Nakhon Sawan | 0.070903 |
| 600501 | Tha Ngio | Nakhon Sawan | 0.124299 |
| 600502 | Bang Ta Ngai | Nakhon Sawan | 0.135579 |
| 600503 | Hu Kwang | Nakhon Sawan | 0.11339 |
| 600504 | Ang Thong | Nakhon Sawan | 0.103499 |
| 600505 | Ban Daen | Nakhon Sawan | 0.098985 |
| 600506 | Bang Kaeo | Nakhon Sawan | 0.102085 |
| 600507 | Ta Khit | Nakhon Sawan | 0.096115 |
| 600508 | Ta Sang | Nakhon Sawan | 0.090419 |
| 600509 | Dan Chang | Nakhon Sawan | 0.077421 |
| 600510 | Nong Krot | Nakhon Sawan | 0.161644 |
| 600511 | Nong Ta Ngu | Nakhon Sawan | 0.076967 |
| 600512 | Bueng Pla Thu | Nakhon Sawan | 0.071965 |
| 600513 | Charoen Phon | Nakhon Sawan | 0.100853 |
| 600601 | Mahapho | Nakhon Sawan | 0.098519 |
| 600602 | Kao Liao | Nakhon Sawan | 0.098841 |
| 600603 | Nong Tao | Nakhon Sawan | 0.07839 |
| 600604 | Khao Din | Nakhon Sawan | 0.132932 |
| 600605 | Hua Dong | Nakhon Sawan | 0.091007 |
| 600701 | Ta Khli | Nakhon Sawan | 0.061948 |
| 600702 | Chong Khae | Nakhon Sawan | 0.064647 |
| 600703 | Chan Sen | Nakhon Sawan | 0.070777 |
| 600704 | Huai Hom | Nakhon Sawan | 0.069991 |
| 600705 | Hua Wai | Nakhon Sawan | 0.073071 |
| 600706 | Nong Pho | Nakhon Sawan | 0.074449 |
| 600707 | Nong Mo | Nakhon Sawan | 0.072871 |
| 600708 | Soi Thong | Nakhon Sawan | 0.072084 |
| 600709 | Lat Thippharot | Nakhon Sawan | 0.070671 |
| 600710 | Phrom Nimit | Nakhon Sawan | 0.068945 |
| 600801 | Tha Tako | Nakhon Sawan | 0.064559 |
| 600802 | Phanom Rok | Nakhon Sawan | 0.07302 |
| 600803 | Hua Thanon | Nakhon Sawan | 0.074955 |
| 600804 | Sai Lamphong | Nakhon Sawan | 0.072973 |
| 600805 | Wang Mahakon | Nakhon Sawan | 0.077949 |
| 600806 | Don Kha | Nakhon Sawan | 0.069403 |
| 600807 | Thamnop | Nakhon Sawan | 0.070168 |
| 600808 | Wang Yai | Nakhon Sawan | 0.07033 |
| 600809 | Phanom Set | Nakhon Sawan | 0.088625 |
| 600810 | Nong Luang | Nakhon Sawan | 0.084722 |
| 600901 | Khok Duea | Nakhon Sawan | 0.070537 |
| 600902 | Samrong Chai | Nakhon Sawan | 0.070705 |
| 600903 | Wang Nam Lat | Nakhon Sawan | 0.070185 |
| 600904 | Takhro | Nakhon Sawan | 0.070106 |
| 600905 | Pho Prasat | Nakhon Sawan | 0.072889 |
| 600906 | Wang Khoi | Nakhon Sawan | 0.071407 |
| 600907 | Na Khom | Nakhon Sawan | 0.070352 |
| 600908 | Phaisali | Nakhon Sawan | 0.067514 |
| 601001 | Phayuha | Nakhon Sawan | 0.099037 |
| 601002 | Noen Makok | Nakhon Sawan | 0.076114 |
| 601003 | Nikhom Khao Bo Kaeo | Nakhon Sawan | 0.07315 |
| 601004 | Muang Hak | Nakhon Sawan | 0.086289 |
| 601005 | Yang Khao | Nakhon Sawan | 0.103564 |
| 601006 | Yan Matsi | Nakhon Sawan | 0.144136 |
| 601007 | Khao Thong | Nakhon Sawan | 0.072018 |
| 601008 | Tha Nam Oi | Nakhon Sawan | 0.104452 |
| 601009 | Nam Song | Nakhon Sawan | 0.12966 |
| 601010 | Khao Kala | Nakhon Sawan | 0.070871 |
| 601011 | Sa Thale | Nakhon Sawan | 0.08202 |
| 601101 | Lat Yao | Nakhon Sawan | 0.069517 |
| 601102 | Huai Nam Hom | Nakhon Sawan | 0.092147 |
| 601103 | Wang Ma | Nakhon Sawan | 0.082465 |
| 601104 | Wang Mueang | Nakhon Sawan | 0.10666 |
| 601105 | Soi Lakhon | Nakhon Sawan | 0.104686 |
| 601106 | Map Kae | Nakhon Sawan | 0.095117 |
| 601107 | Nong Yao | Nakhon Sawan | 0.075814 |
| 601108 | Nong Nom Wua | Nakhon Sawan | 0.070329 |
| 601109 | Ban Rai | Nakhon Sawan | 0.07061 |
| 601110 | Noen Khi Lek | Nakhon Sawan | 0.070336 |
| 601116 | San Chao Kai To | Nakhon Sawan | 0.073278 |
| 601117 | Sa Kaeo | Nakhon Sawan | 0.073596 |
| 601201 | Tak Fa | Nakhon Sawan | 0.065889 |
| 601202 | Lam Phayon | Nakhon Sawan | 0.075405 |
| 601203 | Suk Samran | Nakhon Sawan | 0.078223 |
| 601204 | Nong Phikun | Nakhon Sawan | 0.069971 |
| 601205 | Phu Nok Yung | Nakhon Sawan | 0.075187 |
| 601206 | Udom Thanya | Nakhon Sawan | 0.070692 |
| 601207 | Khao Chai Thong | Nakhon Sawan | 0.0697 |
| 601301 | Mae Wong | Nakhon Sawan | 0.071352 |
| 601303 | Mae Le | Nakhon Sawan | 0.071564 |
| 601304 | Wang San | Nakhon Sawan | 0.077133 |
| 601305 | Khao Chon Kan | Nakhon Sawan | 0.075073 |
| 601401 | Mae Poen | Nakhon Sawan | 0.071136 |
| 601501 | Chum Ta Bong | Nakhon Sawan | 0.077836 |
| 601502 | Pang Sawan | Nakhon Sawan | 0.073084 |
| 610101 | Uthai Mai | Uthai Thani | 0.073502 |
| 610102 | Nam Suem | Uthai Thani | 0.157184 |
| 610103 | Sakae Krang | Uthai Thani | 0.150976 |
| 610104 | Don Khwang | Uthai Thani | 0.108757 |
| 610107 | Tha Sung | Uthai Thani | 0.192964 |
| 610108 | Nong Kae | Uthai Thani | 0.108718 |
| 610109 | Non Lek | Uthai Thani | 0.17191 |
| 610110 | Nong Tao | Uthai Thani | 0.115627 |
| 610111 | Nong Phai Baen | Uthai Thani | 0.162729 |
| 610112 | Nong Phang Kha | Uthai Thani | 0.125292 |
| 610113 | Thung Yai | Uthai Thani | 0.1458 |
| 610114 | Noen Chaeng | Uthai Thani | 0.144302 |
| 610201 | Thapthan | Uthai Thani | 0.111422 |
| 610202 | Thung Na Thai | Uthai Thani | 0.086032 |
| 610203 | Khao Khi Foi | Uthai Thani | 0.101991 |
| 610204 | Nong Ya Plong | Uthai Thani | 0.10906 |
| 610205 | Khok Mo | Uthai Thani | 0.100147 |
| 610206 | Nong Yai Da | Uthai Thani | 0.102949 |
| 610207 | Nong Klang Dong | Uthai Thani | 0.073867 |
| 610208 | Nong Krathum | Uthai Thani | 0.072607 |
| 610209 | Nong Sa | Uthai Thani | 0.077956 |
| 610210 | Taluk Duk | Uthai Thani | 0.079655 |
| 610301 | Sawang Arom | Uthai Thani | 0.119445 |
| 610302 | Nong Luang | Uthai Thani | 0.095238 |
| 610303 | Phluang Song Nang | Uthai Thani | 0.077561 |
| 610304 | Phai Khiao | Uthai Thani | 0.085564 |
| 610305 | Bo Yang | Uthai Thani | 0.093624 |
| 610401 | Nong Chang | Uthai Thani | 0.111546 |
| 610402 | Nong Yang | Uthai Thani | 0.082709 |
| 610403 | Nong Nang Nuan | Uthai Thani | 0.093914 |
| 610404 | Nong Suang | Uthai Thani | 0.097818 |
| 610405 | Ban Kao | Uthai Thani | 0.099805 |
| 610406 | Uthai Kao | Uthai Thani | 0.106106 |
| 610407 | Thung Pho | Uthai Thani | 0.104298 |
| 610408 | Thung Phong | Uthai Thani | 0.086306 |
| 610409 | Khao Bang Kraek | Uthai Thani | 0.083414 |
| 610410 | Khao Kwang Thong | Uthai Thani | 0.077093 |
| 610501 | Nong Kha Yang | Uthai Thani | 0.104863 |
| 610502 | Nong Phai | Uthai Thani | 0.104564 |
| 610503 | Don Kloi | Uthai Thani | 0.164163 |
| 610504 | Huai Rop | Uthai Thani | 0.153587 |
| 610505 | Thung Phueng | Uthai Thani | 0.089329 |
| 610506 | Tha Pho | Uthai Thani | 0.177085 |
| 610507 | Mok Thaeo | Uthai Thani | 0.112979 |
| 610508 | Lum Khao | Uthai Thani | 0.126687 |
| 610509 | Dong Khwang | Uthai Thani | 0.092234 |
| 610601 | Ban Rai | Uthai Thani | 0.073535 |
| 610602 | Thap Luang | Uthai Thani | 0.113727 |
| 610603 | Huai Haeng | Uthai Thani | 0.071262 |
| 610604 | Khok Khwai | Uthai Thani | 0.069954 |
| 610605 | Wang Hin | Uthai Thani | 0.07273 |
| 610606 | Mueang Karung | Uthai Thani | 0.073257 |
| 610607 | Kaen Makrut | Uthai Thani | 0.070648 |
| 610609 | Nong Chok | Uthai Thani | 0.1305 |
| 610610 | Hu Chang | Uthai Thani | 0.076292 |
| 610611 | Ban Bueng | Uthai Thani | 0.081024 |
| 610612 | Ban Mai Khlong Khian | Uthai Thani | 0.072425 |
| 610613 | Nong Bom Kluai | Uthai Thani | 0.08732 |
| 610614 | Chao Wat | Uthai Thani | 0.073658 |
| 610701 | Lan Sak | Uthai Thani | 0.074545 |
| 610702 | Pradu Yuen | Uthai Thani | 0.075071 |
| 610703 | Pa O | Uthai Thani | 0.072397 |
| 610704 | Rabam | Uthai Thani | 0.070674 |
| 610705 | Nam Rop | Uthai Thani | 0.070731 |
| 610706 | Thung Na Ngam | Uthai Thani | 0.073486 |
| 610801 | Suk Ruethai | Uthai Thani | 0.072322 |
| 610802 | Thong Lang | Uthai Thani | 0.069925 |
| 610803 | Huai Khot | Uthai Thani | 0.071069 |
| 620404 | Pa Phutsa | Kamphaeng Phet | 0 |
| 620405 | Saen To | Kamphaeng Phet | 0 |
| 620407 | Bo Tham | Kamphaeng Phet | 0.070216 |
| 620903 | Pang Ta Wai | Kamphaeng Phet | 0 |
| 621002 | Wang Cha-On | Kamphaeng Phet | 0 |
| 621003 | Rahan | Kamphaeng Phet | 0 |
| 630804 | Mae Chan | Tak | 0 |
| 630805 | Mae Lamung | Tak | 0 |
| 660504 | Noen Makok | Phichit | 0 |
| 660505 | Wang Samrong | Phichit | 0 |
| 660507 | Wang Krot | Phichit | 0 |
| 660603 | Thanong | Phichit | 0 |
| 660606 | Tha Khamin | Phichit | 0 |
| 660607 | Tha Sao | Phichit | 0 |
| 660611 | Tha Nang | Phichit | 0 |
| 661001 | Huai Kaeo | Phichit | 0 |
| 661002 | Pho Sai Ngam | Phichit | 0 |
| 661003 | Laem Rang | Phichit | 0 |
| 661101 | Wang Ngio Tai | Phichit | 0 |
| 661103 | Huai Ruam | Phichit | 0 |
| 661104 | Huai Phuk | Phichit | 0 |
| 670205 | Lat Khae | Phetchabun | 0 |
| 670206 | Ban Kluai | Phetchabun | 0 |
| 670506 | Bo Rang | Phetchabun | 0 |
| 670509 | Phu Nam Yot | Phetchabun | 0 |
| 670514 | Sap Noi | Phetchabun | 0 |
| 670602 | Sa Kruat | Phetchabun | 0 |
| 670603 | Khlong Krachang | Phetchabun | 0 |
| 670604 | Na Sanun | Phetchabun | 0 |
| 670607 | Pradu Ngam | Phetchabun | 0 |
| 670805 | Wang Phikun | Phetchabun | 0 |
| 670806 | Phaya Wang | Phetchabun | 0 |
| 670807 | Si Mongkhon | Phetchabun | 0 |
| 700101 | Na Mueang | Ratchaburi | 0.064664 |
| 700102 | Chedi Hak | Ratchaburi | 0.078202 |
| 700103 | Don Tako | Ratchaburi | 0.11013 |
| 700108 | Ang Thong | Ratchaburi | 0.144395 |
| 700109 | Khok Mo | Ratchaburi | 0.171964 |
| 700110 | Sam Ruean | Ratchaburi | 0.18658 |
| 700113 | Don Rae | Ratchaburi | 0.120133 |
| 700114 | Hin Kong | Ratchaburi | 0.127643 |
| 700115 | Khao Raeng | Ratchaburi | 0.138365 |
| 700116 | Ko Phlapphla | Ratchaburi | 0.107764 |
| 700204 | Dan Thap Tako | Ratchaburi | 0.146229 |
| 700205 | Kaem On | Ratchaburi | 0.13245 |
| 700206 | Rang Bua | Ratchaburi | 0.152001 |
| 700301 | Suan Phueng | Ratchaburi | 0.148496 |
| 700302 | Pa Wai | Ratchaburi | 0.13521 |
| 700304 | Tha Khoei | Ratchaburi | 0.142395 |
| 700307 | Tanao Si | Ratchaburi | 0.149815 |
| 700408 | Ban Rai | Ratchaburi | 0.184605 |
| 700501 | Ban Pong | Ratchaburi | 0.112749 |
| 700502 | Tha Pha | Ratchaburi | 0.152567 |
| 700503 | Krap Yai | Ratchaburi | 0.140116 |
| 700504 | Pak Raet | Ratchaburi | 0.092401 |
| 700505 | Nong Kop | Ratchaburi | 0.104331 |
| 700510 | Ban Muang | Ratchaburi | 0.175858 |
| 700511 | Khung Phayom | Ratchaburi | 0.19915 |
| 700512 | Nong Pla Mo | Ratchaburi | 0.118745 |
| 700514 | Boek Phrai | Ratchaburi | 0.162235 |
| 700515 | Lat Bua Khao | Ratchaburi | 0.195803 |
| 700602 | Wang Yen | Ratchaburi | 0.116993 |
| 700702 | Don Krabueang | Ratchaburi | 0.105251 |
| 700703 | Nong Pho | Ratchaburi | 0.165176 |
| 700704 | Ban Lueak | Ratchaburi | 0.16603 |
| 700716 | Nang Kaeo | Ratchaburi | 0.14624 |
| 700717 | Thammasen | Ratchaburi | 0.176262 |
| 700805 | Pak Tho | Ratchaburi | 0.160652 |
| 700806 | Pa Kai | Ratchaburi | 0.088401 |
| 700807 | Wat Yang Ngam | Ratchaburi | 0.175706 |
| 700808 | Ang Hin | Ratchaburi | 0.150406 |
| 700809 | Bo Kradan | Ratchaburi | 0.158263 |
| 700810 | Yang Hak | Ratchaburi | 0.168284 |
| 700811 | Wan Dao | Ratchaburi | 0.120795 |
| 701001 | Ban Kha | Ratchaburi | 0.145391 |
| 701002 | Ban Bueng | Ratchaburi | 0.143352 |
| 701003 | Nong Phan Chan | Ratchaburi | 0.147393 |
| 710101 | Ban Nuea | Kanchanaburi | 0.062896 |
| 710102 | Ban Tai | Kanchanaburi | 0.125357 |
| 710103 | Pak Phraek | Kanchanaburi | 0.130206 |
| 710104 | Tha Makham | Kanchanaburi | 0.157638 |
| 710105 | Kaeng Sian | Kanchanaburi | 0.129207 |
| 710106 | Nong Bua | Kanchanaburi | 0.123606 |
| 710107 | Lat Ya | Kanchanaburi | 0.116923 |
| 710108 | Wang Dong | Kanchanaburi | 0.149689 |
| 710109 | Chong Sadao | Kanchanaburi | 0.14931 |
| 710110 | Nong Ya | Kanchanaburi | 0.179381 |
| 710111 | Ko Samrong | Kanchanaburi | 0.167665 |
| 710113 | Ban Kao | Kanchanaburi | 0.139005 |
| 710116 | Wang Yen | Kanchanaburi | 0.185281 |
| 710201 | Lum Sum | Kanchanaburi | 0.165134 |
| 710202 | Tha Sao | Kanchanaburi | 0.156735 |
| 710203 | Sing | Kanchanaburi | 0.128378 |
| 710204 | Sai Yok | Kanchanaburi | 0.143145 |
| 710205 | Wang Krachae | Kanchanaburi | 0.156097 |
| 710206 | Si Mongkhon | Kanchanaburi | 0.145894 |
| 710207 | Bong Ti | Kanchanaburi | 0.143155 |
| 710301 | Bo Phloi | Kanchanaburi | 0.12438 |
| 710302 | Nong Kum | Kanchanaburi | 0.10136 |
| 710303 | Nong Ri | Kanchanaburi | 0.13981 |
| 710305 | Lum Rang | Kanchanaburi | 0.103271 |
| 710308 | Chong Dan | Kanchanaburi | 0.116571 |
| 710309 | Nong Krang | Kanchanaburi | 0.098556 |
| 710401 | Na Suan | Kanchanaburi | 0.145248 |
| 710402 | Dan Mae Chalaep | Kanchanaburi | 0.144687 |
| 710403 | Nong Pet | Kanchanaburi | 0.140986 |
| 710404 | Tha Kradan | Kanchanaburi | 0.142991 |
| 710405 | Khao Chot | Kanchanaburi | 0.142985 |
| 710406 | Mae Krabung | Kanchanaburi | 0.140737 |
| 710502 | Yang Muang | Kanchanaburi | 0.088935 |
| 710503 | Don Cha-Em | Kanchanaburi | 0.088335 |
| 710504 | Tha Mai | Kanchanaburi | 0.154872 |
| 710505 | Takhram En | Kanchanaburi | 0.122933 |
| 710506 | Tha Maka | Kanchanaburi | 0.128828 |
| 710507 | Tha Ruea | Kanchanaburi | 0.100583 |
| 710508 | Khok Tabong | Kanchanaburi | 0.135908 |
| 710510 | Ulok Si Muen | Kanchanaburi | 0.100851 |
| 710511 | Khao Sam Sip Hap | Kanchanaburi | 0.153681 |
| 710512 | Phra Thaen | Kanchanaburi | 0.057549 |
| 710513 | Wai Niao | Kanchanaburi | 0.193322 |
| 710514 | Saen To | Kanchanaburi | 0.191442 |
| 710515 | Sanam Yae | Kanchanaburi | 0.118185 |
| 710516 | Tha Sao | Kanchanaburi | 0.172786 |
| 710517 | Nong Lan | Kanchanaburi | 0.091877 |
| 710601 | Tha Muang | Kanchanaburi | 0.105438 |
| 710602 | Wang Khanai | Kanchanaburi | 0.146461 |
| 710603 | Wang Sala | Kanchanaburi | 0.147317 |
| 710604 | Tha Lo | Kanchanaburi | 0.175891 |
| 710605 | Nong Khao | Kanchanaburi | 0.088862 |
| 710606 | Thung Thong | Kanchanaburi | 0.164155 |
| 710607 | Khao Noi | Kanchanaburi | 0.141585 |
| 710608 | Muang Chum | Kanchanaburi | 0.184528 |
| 710609 | Ban Mai | Kanchanaburi | 0.12899 |
| 710610 | Phang Tru | Kanchanaburi | 0.134663 |
| 710612 | Rang Sali | Kanchanaburi | 0.136042 |
| 710613 | Nong Tak Ya | Kanchanaburi | 0.147632 |
| 710701 | Tha Khanun | Kanchanaburi | 0.156607 |
| 710702 | Pi Lok | Kanchanaburi | 0.144972 |
| 710703 | Hin Dat | Kanchanaburi | 0.141206 |
| 710704 | Lin Thin | Kanchanaburi | 0.144814 |
| 710705 | Chalae | Kanchanaburi | 0.139868 |
| 710706 | Huai Khayeng | Kanchanaburi | 0.147351 |
| 710707 | Sahakon Nikhom | Kanchanaburi | 0.145846 |
| 710801 | Nong Lu | Kanchanaburi | 0.14471 |
| 710802 | Prang Phle | Kanchanaburi | 0.147033 |
| 710803 | Lai Wo | Kanchanaburi | 0.139549 |
| 710901 | Phanom Thuan | Kanchanaburi | 0.135229 |
| 710902 | Nong Rong | Kanchanaburi | 0.103485 |
| 710903 | Thung Samo | Kanchanaburi | 0.103671 |
| 710904 | Don Chedi | Kanchanaburi | 0.170299 |
| 710905 | Phang Tru | Kanchanaburi | 0.132563 |
| 710906 | Rang Wai | Kanchanaburi | 0.139557 |
| 710911 | Nong Sarai | Kanchanaburi | 0.105732 |
| 710912 | Don Ta Phet | Kanchanaburi | 0.100637 |
| 711001 | Lao Khwan | Kanchanaburi | 0.125636 |
| 711002 | Nong Sano | Kanchanaburi | 0.16314 |
| 711003 | Nong Pradu | Kanchanaburi | 0.090768 |
| 711005 | Nong Nok Kaeo | Kanchanaburi | 0.091506 |
| 711006 | Thung Krabam | Kanchanaburi | 0.106152 |
| 711101 | Dan Makham Tia | Kanchanaburi | 0.130754 |
| 711102 | Klon Do | Kanchanaburi | 0.134505 |
| 711103 | Chorakhe Phueak | Kanchanaburi | 0.133585 |
| 711104 | Nong Phai | Kanchanaburi | 0.121426 |
| 711201 | Nong Prue | Kanchanaburi | 0.136429 |
| 711202 | Nong Pla Lai | Kanchanaburi | 0.107997 |
| 711203 | Somdet Charoen | Kanchanaburi | 0.13479 |
| 711301 | Huai Krachao | Kanchanaburi | 0.103259 |
| 711302 | Wang Phai | Kanchanaburi | 0.119608 |
| 711303 | Don Salaep | Kanchanaburi | 0.115344 |
| 711304 | Sa Long Ruea | Kanchanaburi | 0.14357 |
| 720204 | Khao Din | Suphan Buri | 0.136116 |
| 720206 | Thung Khli | Suphan Buri | 0.116027 |
| 720207 | Khok Chang | Suphan Buri | 0.115959 |
| 720209 | Hua Na | Suphan Buri | 0.153272 |
| 720210 | Bo Kru | Suphan Buri | 0.159327 |
| 720212 | Pa Sakae | Suphan Buri | 0.180989 |
| 720213 | Yang Non | Suphan Buri | 0.174432 |
| 720304 | Ong Phra | Suphan Buri | 0.150692 |
| 720307 | Wang Yao | Suphan Buri | 0.146013 |
| 720604 | Sa Krachom | Suphan Buri | 0.187557 |
| 720605 | Thale Bok | Suphan Buri | 0.173084 |
| 720712 | Thung Khok | Suphan Buri | 0.135182 |
| 720713 | Nong Bo | Suphan Buri | 0.138686 |
| 720714 | Bo Suphan | Suphan Buri | 0.126162 |
| 720715 | Don Manao | Suphan Buri | 0.187232 |
| 720802 | Wang Luek | Suphan Buri | 0.172557 |
| 720804 | Nong Phak Nak | Suphan Buri | 0.195765 |
| 720806 | Nong Sadao | Suphan Buri | 0.173619 |
| 720901 | U Thong | Suphan Buri | 0.183216 |
| 720902 | Sa Yai Som | Suphan Buri | 0.162866 |
| 720903 | Chorakhe Sam Phan | Suphan Buri | 0.178457 |
| 720907 | Nong Ong | Suphan Buri | 0.155278 |
| 720909 | Phlapphla Chai | Suphan Buri | 0.169408 |
| 720910 | Ban Khong | Suphan Buri | 0.182408 |
| 721001 | Nong Ya Sai | Suphan Buri | 0.110496 |
| 721002 | Nong Ratchawat | Suphan Buri | 0.120992 |
| 721003 | Nong Pho | Suphan Buri | 0.152862 |
| 730101 | Phra Pathom Chedi | Nakhon Pathom | 0.043987 |
| 730104 | Thammasala | Nakhon Pathom | 0.16145 |
| 730105 | Ta Kong | Nakhon Pathom | 0.195946 |
| 730106 | Map Khae | Nakhon Pathom | 0.158644 |
| 730107 | Sanam Chan | Nakhon Pathom | 0.115635 |
| 730109 | Thanon Khat | Nakhon Pathom | 0.174452 |
| 730111 | Nakhon Pathom | Nakhon Pathom | 0.081056 |
| 730117 | Wang Yen | Nakhon Pathom | 0.180475 |
| 730119 | Lam Phaya | Nakhon Pathom | 0.129316 |
| 730121 | Suan Pan | Nakhon Pathom | 0.185217 |
| 730122 | Huai Chorakhe | Nakhon Pathom | 0.025395 |
| 730125 | Ban Yang | Nakhon Pathom | 0.132776 |
| 730202 | Kratip | Nakhon Pathom | 0.109914 |
| 730205 | Thung Khwang | Nakhon Pathom | 0.165213 |
| 730207 | Thung Bua | Nakhon Pathom | 0.121012 |
| 730210 | Huai Mon Thong | Nakhon Pathom | 0.15627 |
| 730212 | Kamphaeng Saen | Nakhon Pathom | 0.133453 |
| 730213 | Rang Phikun | Nakhon Pathom | 0.123051 |
| 730214 | Nong Krathum | Nakhon Pathom | 0.101453 |
| 730301 | Nakhon Chai Si | Nakhon Pathom | 0.180967 |
| 730302 | Bang Krabao | Nakhon Pathom | 0.10038 |
| 730303 | Wat Khae | Nakhon Pathom | 0.185083 |
| 730304 | Tha Tamnak | Nakhon Pathom | 0.118052 |
| 730305 | Bang Kaeo | Nakhon Pathom | 0.170165 |
| 730308 | Tha Phraya | Nakhon Pathom | 0.193179 |
| 730309 | Phaniat | Nakhon Pathom | 0.05284 |
| 730310 | Bang Rakam | Nakhon Pathom | 0.129093 |
| 730311 | Khok Phra Chedi | Nakhon Pathom | 0.187016 |
| 730312 | Sisa Thong | Nakhon Pathom | 0.140179 |
| 730317 | Don Faek | Nakhon Pathom | 0.186011 |
| 730604 | Bang Krathuek | Nakhon Pathom | 0.193686 |
| 730609 | Tha Talat | Nakhon Pathom | 0.198989 |
| 730611 | Khlong Mai | Nakhon Pathom | 0.179603 |
| 730613 | Khlong Chinda | Nakhon Pathom | 0.182489 |
| 730616 | Om Yai | Nakhon Pathom | 0.136963 |
| 730701 | Sala Ya | Nakhon Pathom | 0.14884 |
| 730703 | Mahasawat | Nakhon Pathom | 0.110745 |
| 740101 | Mahachai | Samut Sakhon | 0.131442 |
| 740102 | Tha Chalom | Samut Sakhon | 0.174606 |
| 740109 | Na Di | Samut Sakhon | 0.136622 |
| 740111 | Khok Krabue | Samut Sakhon | 0.168993 |
| 740113 | Phanthai Norasing | Samut Sakhon | 0.191901 |
| 740114 | Khok Kham | Samut Sakhon | 0.154915 |
| 740117 | Bang Ya Phraek | Samut Sakhon | 0.191427 |
| 740202 | Om Noi | Samut Sakhon | 0.089169 |
| 740204 | Suan Luang | Samut Sakhon | 0.136647 |
| 740206 | Khlong Maduea | Samut Sakhon | 0.110124 |
| 740208 | Don Kai Di | Samut Sakhon | 0.19808 |
| 740209 | Khae Rai | Samut Sakhon | 0.199509 |
| 750310 | Phraek Nam Daeng | Samut Songkhram | 0.138774 |
| 750311 | Yi San | Samut Songkhram | 0.160744 |
| 760101 | Tha Rap | Phetchaburi | 0.14706 |
| 760102 | Khlong Krachaeng | Phetchaburi | 0.076379 |
| 760103 | Bang Chan | Phetchaburi | 0.12909 |
| 760104 | Na Phan Sam | Phetchaburi | 0.098283 |
| 760105 | Thong Chai | Phetchaburi | 0.168549 |
| 760108 | Rai Som | Phetchaburi | 0.130481 |
| 760109 | Wiang Khoi | Phetchaburi | 0.10324 |
| 760110 | Bang Chak | Phetchaburi | 0.143306 |
| 760111 | Ban Mo | Phetchaburi | 0.131032 |
| 760112 | Ton Mamuang | Phetchaburi | 0.120077 |
| 760113 | Chong Sakae | Phetchaburi | 0.128497 |
| 760114 | Na Wung | Phetchaburi | 0.158105 |
| 760115 | Sam Marong | Phetchaburi | 0.11273 |
| 760116 | Pho Phra | Phetchaburi | 0.134574 |
| 760117 | Hat Chao Samran | Phetchaburi | 0.144383 |
| 760118 | Hua Saphan | Phetchaburi | 0.116712 |
| 760119 | Ton Maphrao | Phetchaburi | 0.117367 |
| 760120 | Wang Tako | Phetchaburi | 0.101248 |
| 760121 | Pho Rai Wan | Phetchaburi | 0.148809 |
| 760122 | Don Yang | Phetchaburi | 0.140839 |
| 760123 | Nong Khanan | Phetchaburi | 0.14532 |
| 760124 | Nong Phlap | Phetchaburi | 0.10779 |
| 760201 | Khao Yoi | Phetchaburi | 0.157195 |
| 760202 | Sa Phang | Phetchaburi | 0.151023 |
| 760203 | Bang Khem | Phetchaburi | 0.136151 |
| 760204 | Thap Khang | Phetchaburi | 0.170362 |
| 760205 | Nong Pla Lai | Phetchaburi | 0.179954 |
| 760206 | Nong Prong | Phetchaburi | 0.122496 |
| 760207 | Nong Chumphon | Phetchaburi | 0.138565 |
| 760208 | Huai Rong | Phetchaburi | 0.144755 |
| 760209 | Huai Tha Chang | Phetchaburi | 0.127594 |
| 760302 | Yang Nam Klat Nuea | Phetchaburi | 0.156543 |
| 760303 | Yang Nam Klat Tai | Phetchaburi | 0.143218 |
| 760401 | Cha-Am | Phetchaburi | 0.138185 |
| 760402 | Bang Kao | Phetchaburi | 0.147755 |
| 760403 | Na Yang | Phetchaburi | 0.146965 |
| 760404 | Khao Yai | Phetchaburi | 0.142213 |
| 760405 | Nong Sala | Phetchaburi | 0.124916 |
| 760406 | Huai Sai Nuea | Phetchaburi | 0.143555 |
| 760407 | Rai Mai Phatthana | Phetchaburi | 0.144379 |
| 760408 | Sam Phraya | Phetchaburi | 0.136208 |
| 760409 | Don Khun Huai | Phetchaburi | 0.187487 |
| 760501 | Tha Yang | Phetchaburi | 0.179491 |
| 760504 | Nong Chok | Phetchaburi | 0.140819 |
| 760505 | Map Pla Khao | Phetchaburi | 0.167525 |
| 760506 | Tha Mai Ruak | Phetchaburi | 0.187743 |
| 760507 | Wang Khrai | Phetchaburi | 0.193659 |
| 760511 | Klat Luang | Phetchaburi | 0.177567 |
| 760512 | Puek Tian | Phetchaburi | 0.158318 |
| 760513 | Khao Krapuk | Phetchaburi | 0.158786 |
| 760514 | Tha Laeng | Phetchaburi | 0.197475 |
| 760515 | Ban Nai Dong | Phetchaburi | 0.156419 |
| 760602 | Ban Hat | Phetchaburi | 0.165831 |
| 760603 | Ban Than | Phetchaburi | 0.142565 |
| 760606 | Rai Makham | Phetchaburi | 0.184198 |
| 760608 | Nong Krachet | Phetchaburi | 0.154143 |
| 760609 | Nong Kapu | Phetchaburi | 0.143112 |
| 760610 | Lat Pho | Phetchaburi | 0.143897 |
| 760611 | Saphan Krai | Phetchaburi | 0.114647 |
| 760612 | Rai Khok | Phetchaburi | 0.137773 |
| 760613 | Rong Khe | Phetchaburi | 0.147833 |
| 760614 | Rai Sathon | Phetchaburi | 0.151501 |
| 760615 | Huai Khong | Phetchaburi | 0.143422 |
| 760618 | Huai Luek | Phetchaburi | 0.161609 |
| 760701 | Ban Laem | Phetchaburi | 0.187981 |
| 760702 | Bang Khun Sai | Phetchaburi | 0.164837 |
| 760703 | Pak Thale | Phetchaburi | 0.176595 |
| 760704 | Bang Kaeo | Phetchaburi | 0.168662 |
| 760705 | Laem Phak Bia | Phetchaburi | 0.150549 |
| 760709 | Tha Raeng | Phetchaburi | 0.191428 |
| 760710 | Tha Raeng Ok | Phetchaburi | 0.19845 |
| 760801 | Kaeng Krachan | Phetchaburi | 0.168069 |
| 760802 | Song Phi Nong | Phetchaburi | 0.176317 |
| 760803 | Wang Chan | Phetchaburi | 0.188904 |
| 760804 | Pa Deng | Phetchaburi | 0.152119 |
| 760805 | Phu Sawan | Phetchaburi | 0.199838 |
| 760806 | Huai Mae Phriang | Phetchaburi | 0.142259 |
| 770701 | Hua Hin | Prachuap Khiri Khan | 0 |
| 770703 | Hin Lek Fai | Prachuap Khiri Khan | 0 |
| 770704 | Nong Phlap | Prachuap Khiri Khan | 0 |
| 770706 | Huai Sat Yai | Prachuap Khiri Khan | 0 |
